# Chronic dental lighting disrupts blood-retinal barrier homeostasis via vascular and inflammatory pathways

**DOI:** 10.1038/s41368-025-00414-3

**Published:** 2026-02-13

**Authors:** Haiyang Sun, Shuhuai Meng, He Cai, Zhengyi Xu, Kuo Gai, Dan Meng, Yixin Shi, Feng Luo, Xibo Pei, Jian Wang, Anjali P. Kusumbe, Qianbing Wan, Junyu Chen

**Affiliations:** 1 State Key Laboratory of Oral Diseases, National Clinical Research Center for Oral Diseases, Department of Prosthodontics, West China Hospital of Stomatology, Sichuan University, Chengdu, China; 2 West China Hospital, Sichuan University, Chengdu, China; 3 Department of Stomatology, West China Tianfu Hospital, Sichuan University, Chengdu, China; 4 Department of Ophthalmology, West China Hospital, Sichuan University, Chengdu, China; 5 Tissue and Tumor Microenvironments Lab, Cancer Discovery and Regenerative Medicine Program, Lee Kong Chian School of Medicine, Nanyang Technological University, Singapore, Singapore; 6 Multidisciplinary Institute of Ageing (MIA-Portugal), Coimbra, Portugal

**Keywords:** Risk factors, Cell death

## Abstract

Excessive lighting is integral to dentists’ daily routines but can impair their vision, affecting personal and professional performance. Most studies focus on acute photodamage, neglecting chronic photo-injury from dental lighting and its impact on the blood-retinal barrier homeostasis. An epidemiological survey involving 14,523 individuals showed dentists had 3.6 times higher odds of vision-related issues compared to other occupations (OR = 3.639, 95% CI: 3.064–4.323). Subsequently, chronic photodamage models in rats were created to accurately simulate dental working conditions. Using systematic imaging and gene analysis, including OCT, tissue clearing technology and RNA-sequencing, dental lighting was found to disrupted both inner and outer blood-retinal barriers, reduced retinal blood vessels, and promoted perivascular macrophage recruitment. Among them, the number of capillary branches decreased sharply. Moreover, the activation of inflammatory-related pathways such as NF-κB signaling resulted in the damage of vision-related functional structures in the retina. Notably, among three dental light sources, low-intensity halogen caused minimal retinal damage, whereas blue and white LEDs significantly disrupted blood-retinal barrier homeostasis. This study explored the potential mechanism of dental lighting environment inducing the disruption of blood-retinal barrier homeostasis, and provided essential guidance for dental professionals in selecting light sources, which is conducive to reducing the risk of occupational ocular diseases among dentists.

## Introduction

Excessive illumination is one of the critical risk factors for retina-related diseases, including age-related macular degeneration, degeneration of retinal pigment epithelium (RPE), and glaucoma.^1–3^ The daily work of dental professionals relies heavily on abundant lighting assistance. Moreover, dentists work for prolonged periods under intense illumination. Epidemiological data reveal that dental practitioners have a significantly higher prevalence of ocular diseases than other professionals.^4,5^ These ocular diseases result in visual impairments that have a significant detrimental effect on dentists’ quality of life and impose a considerable economic burden.^6–8^

Large population and animal studies have identified excessive light exposure as a factor contributing to visual problems, resulting in retinal damage and the induction of apoptosis.^9–11^ Most studies have focused on the impact of acute exposures to high luminance or lighting systems with extreme color temperatures, using them as models for light-induced retinal degeneration.^12–14^ However, dental professionals often encounter a work environment contributing to long-term and chronic photodamage, and there is limited understanding of the long-term effects of chronic photodamage of the retina caused by low-intensity light sources.^15,16^ Moreover, current studies have primarily focused on investigating the changes in retinal cell structure induced by light,^17,18^ with limited studies on alterations in the retinal vascular microenvironment. The maintenance of the retinal microenvironment relies heavily on the integrity of the outer blood-retinal barrier composed of retinal pigment epithelial cells and the inner blood-retinal barrier formed by endothelial cells lining the retinal microvasculature.^19^ Disruption of the blood-retinal barrier leads to retinal ischemia, cellular stress, and edema, which play central roles in developing retinal diseases, including age-related macular degeneration and diabetic retinopathy.^20,21^ Therefore, while epidemiological studies reveal a high incidence of ocular diseases among dentists,^5,22–24^ there is a lack of specific model to simulate the chronic light-damaging environment faced by dental practitioners, as well as a scarcity of reliable experimental data on how this chronic photodamage environment affects the blood-retinal barrier homeostasis.

We conducted an analysis and simulation of the lighting patterns used by dentists. Specifically, experimental light models were established by subjecting seven groups of Sprague-Dawley rats to different chronic photodamage patterns for up to 6 months. The observation of the blood-retinal barrier plays a crucial role in understanding the impact of chronic photodamage on the eye. While there are some researches on retinal vasculature,^25–27^ determining the complete three-dimensional state of the blood‒retinal barrier structure remains challenging due to potential alterations caused by prolonged light exposure, affecting its overall structure and texture. The current retinal vascular imaging techniques still have multiple limitations, including prolonged sample preparation time and difficulty in maintaining the three-dimensional topological structure of the retina.^28–30^ Thus, we developed a modified whole-organ tissue clearing and imaging method for the eyeball and retinal tissue that effectively preserved the three-dimensional structure of the retinal vascular network and streamlined the sample processing procedure.

In this study, we conducted an epidemiological survey and investigated the physical properties of light sources commonly used by dentists. Subsequently, a chronic photodamage model in rats was established to simulate the dental operatory environment and investigate the alterations in the blood-retinal barrier stability using comprehensive imaging and gene analysis. This study uniquely combines epidemiological data with a novel tissue clearing technique to model chronic photodamage, addressing gaps in understanding how dental lighting affects retinal vascular homeostasis. Further, we explored the mechanisms of retinal lesions induced by chronic photodamage under dental lighting patterns, hoping to reduce vision-related occupational diseases in the future.

## Results

### High incidence of visual abnormalities among dentists and potential risks of dental light sources

**Fig. 1:**
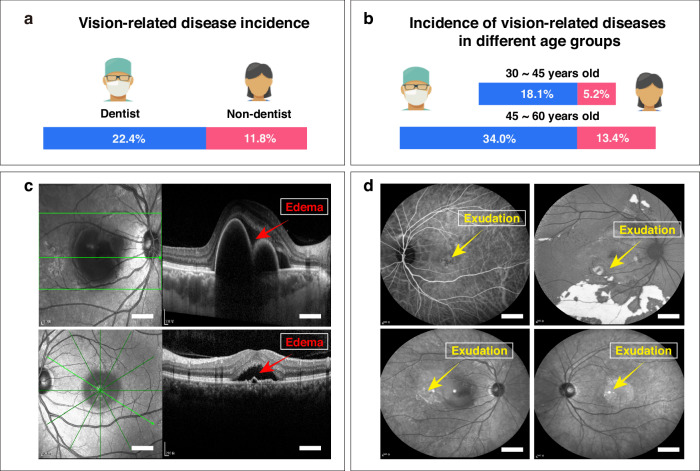
Investigating dentist vision-health and lighting conditions. **a** Bar chart comparing the incidence rates of visual function abnormalities between dentist (shown in blue) and non-dentist (shown in pink). **b** Bar chart comparing the incidence rates of visual abnomalies among different age groups of dentists (shown in blue) and non-dentist (shown in pink). **c** Representative OCT slice images displaying retinal pathology from a dentist. The areas indicated by red arrows correspond to regions of retinal edema. **d** Representative indocyanine green angiography images illustrating retinal pathology from a dentist. The regions indicated by yellow arrows correspond to exudation. Scale bars: (**c**, **d**) 600 μm

To investigate vision-related issues among dentists, we conducted a cross-sectional study based on individuals from the Physical Examination Center, West China Hospital, Sichuan University. This study encompassed 14 523 individuals who underwent physical examinations from January 1, 2021, to January 1, 2022. Among them, 1 140 individuals were dental practitioners. Our findings revealed a higher prevalence of vision-related health issues among dentists, with the rate of 22.4%, compared to 11.8% among non-dentists (Fig. 1a). According to logistic regression, dentists exhibited 3.6-fold higher odds of vision-related issues compared to non-dentists (OR = 3.639, 95% CI: 3.064–4.323, Supplementary Fig. 1a). While participants of 45–60 years had 2.7-time greater odds of vision-related problems than those aged 30–45 years (OR = 2.672, 95% CI: 2.294–3.112, Supplementary Fig. 1a). Consistent with our findings, previous studies have indicated a 15–53% prevalence rate of visual problems among dentists.^22–24^ In contrast, epidemiological data from the past three decades have shown that the incidence of visual impairment in the general population is below 3%.^31^ Upon further analysis, we noted that the incidence rates of retinopathy among dentists in the 30–45 and 45–60 age groups were 18.1% and 34.0% (Fig. 1b), respectively, which were higher compared to the 5.2% and 13.4% rates in the non-dentist population (Fig. 1b). In addition, optical coherence tomography (OCT) examinations revealed significant retinal tissue edema in the macular area a subset of dentists (Fig. 1c and Supplementary Fig. 1b). Indocyanine green angiography on these dentists with abnormal OCT results showed abnormalities in the macular area, including transparent fluorescence and exudation (Fig. 1d and Supplementary Fig. 1b).

**Fig. 2:**
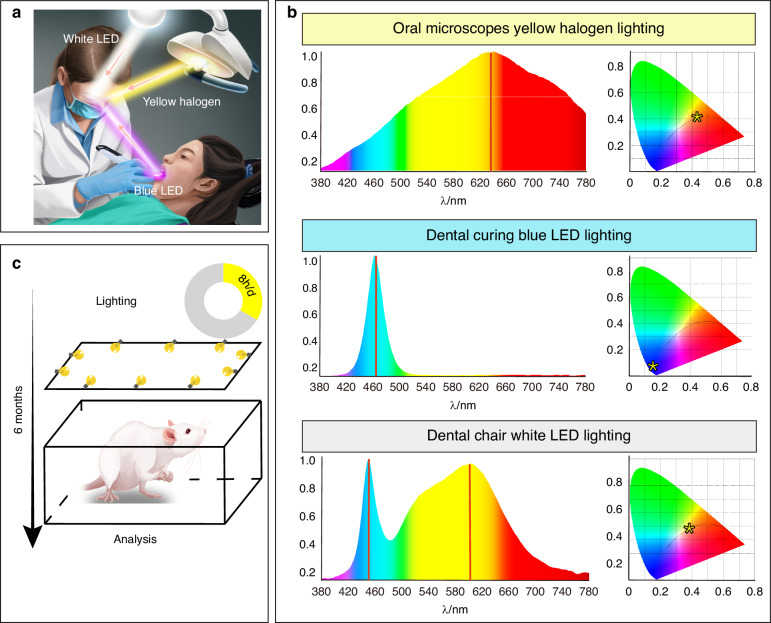
Dental work lighting conditions. **a** Schematic illustration of three types of light sources commonly used by dentists. **b** Photometric characterization results of various light sources. Left panel displays the spectral output of the light sources, with wavelength on the *x*-axis and spectral peak height on the *y*-axis. The red line indicates the peak wavelength of each light source. The right panel shows the chromaticity results of the light sources. The *x*-axis represents the proportion of red primary color, while the *y*-axis represents the proportion of green primary color. Yellow asterisks denote the position of each light source’s color on the chromaticity diagram. **c** Schematic illustration of the dental photodamage model in rats

Based on statistical analysis of the average daily working hours of dentists at West China Hospital of Stomatology, dentists spend an average of approximately 7.36 h per workday engaged in clinical procedures under various lighting conditions. To accurately investigate the relationship between the dental operatory environment and the occurrence of abnormal visual function, we first analyzed the light sources commonly used by dental professionals for an accurate simulation of their working conditions. Dentists commonly use three types of light sources: dental curing lights equipped with blue-light LEDs, dental microscopes with yellow-light halogen lights, and dental chair lighting with white-light LEDs (Fig. 2a).^32–34^ The light which dentists are exposed to mainly comes from two sources (Supplementary Fig. 1c): (1) direct irradiation of light emitted by the equipment mentioned above; (2) indirect irradiation of light reflected through an oral mirror or soft and hard tissues.

Three classic optical parameters, i.e., wavelength, illuminance, and color temperature, were used to analyze the commonly used dental light sources. First, we measured the wavelengths and color temperatures of the three aforementioned light sources. The halogen light source commonly used by dentists exhibited a peak at 630 nm, with a color temperature of around 3 200 K (Fig. 2b and Supplementary Table 1). The dental white LED light source exhibited peaks at 450 and 610 nm (Fig. 2b). Despite having a color temperature of approximately 5 000 K (Supplementary Table 1), the white LED light source contained a mixture of blue light components with a 460-nm wavelength (Fig. 2b). In addition, we examined the illuminance of these light sources through two main pathways: direct entry into the eyes and reflection from the oral mirror (Supplementary Fig. 1c), which exhibited 1 152 ± 113 - 1 552 ± 323 Lux and 212 ± 27–241 ± 56 Lux, respectively (Supplementary Table 1). To reflect the effective radiation exposure of organisms, we also measured spectral irradiance (Supplementary Table 1). The spectral irradiance of the halogen light source ranged from (2.1 ± 0.4) to (20.3 ± 5.9) mW/m²/nm, while that of the blue LED light source varied between (16.8 ± 7.3) and (480.3 ± 17.9) mW/m²/nm. For the white LED light source, the blue component exhibited a spectral irradiance of (4.8 ± 0.5) to (24.1 ± 7.0) mW/m²/nm, and the yellow component ranged from (3.8 ± 0.3) to (12.2 ± 4.3) mW/m²/nm. Based on these parameters, we selected three light sources with corresponding wavelengths, color temperatures, illuminance, and spectral irradiance to establish a light-induced damage model in rats simulating the dental work environment. To simulate dentists’ long-term working conditions, rats were exposed to light 8 h a day, 5 days a week, for 6 months, mimicking the cumulative light load over time (Fig. 2c). Seven groups were established: (a) blank control group without additional light exposure, (b) 200 Lux halogen light group, (c) 1 000 Lux halogen light group, (d) 200 Lux blue LED group, (e) 1 000 Lux blue LED group, (f) 200 Lux white LED group, and (g) 1 000 Lux white LED group.

### The outer blood-retinal barriers were disrupted, and the signals of senescence markers increased under the simulated dental lighting patterns

**Fig. 3:**
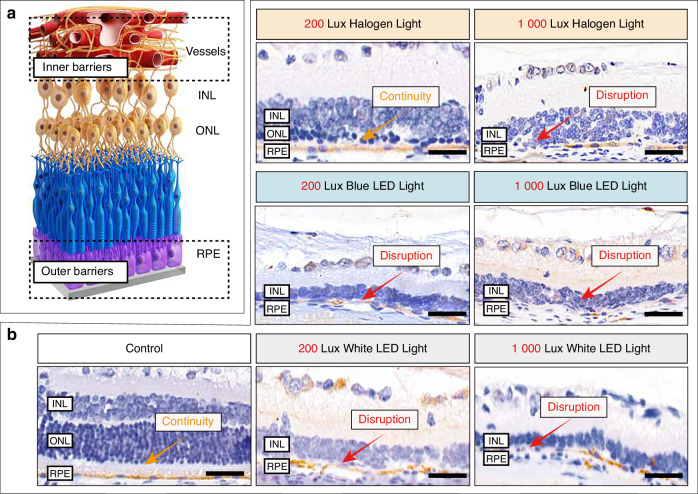
Effects of the simulated dental lighting patterns on the outer blood-retinal barrier after 6-month exposure. **a** Schematic representation of blood-retinal barrier. **b** Representative immunohistochemistry images showing regions of retinal pigment epithelium (RPE). RPE was stained with RDH5. Yellow arrows indicate the continuous RPE layer, and red arrows indicate the disruption of the RPE layer. Scale bars: (**b**) 50 μm

The blood-retinal barrier is essential for maintaining the internal balance and functionality of the retina and serves as a crucial biological barrier in the body. One significant component of this barrier is the outer blood-retinal barrier, formed by the tight junctions between the cells of the retinal pigment epithelium (RPE) (Fig. 3a). This intricate arrangement of tight junctions regulates the movements of substances and prevents the entry of potentially harmful agents, contributing to the preservation of retinal health and homeostasis.^35^ After 6 months of exposure to light at 200 Lux intensity, the RPE continuity was significantly disrupted under blue and white LED lighting (Fig. 3b and Supplementary Fig. 2). However, the RPE maintained its integrity in both the halogen light and control groups (Fig. 3b and Supplementary Fig. 2). When the illumination intensity increased to 1 000 Lux, the RPE layers degenerated and even disappeared under all three light sources (Fig. 3b and Supplementary Fig. 2).

Lipofuscin is a byproduct of retinal aging and tends to accumulate within RPE cells over time. Short-wavelength light can stimulate lipofuscin in RPE cells, resulting in the production of autofluorescence.^36^ Detailed observations of retinal changes were conducted at intervals of 1 month, 3 months, and 6 months. The results revealed a distinct time dependent escalation in retinal injury. After 1 month of light exposure, only the 1 000 Lux blue LED light group exhibited significant fluorescent signals (Supplementary Fig. 3). At the 3-month time point, retinal damage progressively worsened in all groups except the 200 Lux halogen light group, which displayed no significant progression (Supplementary Fig. 3). After 6 months, with the exception of the control group, the 200 Lux halogen light group exhibited the smallest area of high-intensity fluorescence (Supplementary Figs. 3 and 4a, b). Moreover, at a light intensity of 1 000 Lux, there was no significant difference in the area of high-intensity fluorescent signals between the different light sources (Supplementary Figs. 3 and 4a, b).

Next, we explored the structural changes within the inner retina. In a healthy retina, the inner nuclear layer (INL) and outer nuclear layer (ONL) are distinctly layered, positioned between the inner and outer blood-retinal barriers (Fig. 3a). They are crucial for visual processing and light perception.^37^ However, most simulated dental lighting patterns disrupt these structures. The HE results revealed that under the 200 Lux illumination, the cell nuclei exhibited varying degrees of swelling when exposed to the three different light sources (Supplementary Fig. 4c). Additionally, the boundary between the ONL and INL became unclear (Supplementary Fig. 4c). Under 200 Lux illumination, compared to the blue and white LED light groups, the halogen light group exhibited milder pathological changes; under the 1 000 Lux illumination, all three light source groups caused retinal structural changes, with cell nuclei undergoing fragmentation or pyknosis (Supplementary Fig. 5a, b). Particularly, the retinal tissue in the 1 000 Lux blue LED group exhibited remarkable tissue edema (Supplementary Fig. 4c). OCT results confirmed this retinal cell structures destruction (Supplementary Fig. 4c).

Based on these findings, after 6 months of simulated dental work environment, all three commonly used dental light sources resulted in varying degrees of negative impact on the outer blood-retinal barrier. It is worth noting that the 200 Lux halogen light demonstrated slight chronic damage to the outer blood-retinal barrier. However, when the light intensity increased to 1 000 Lux, all light sources disrupted the outer barrier and accumulated senescence markers.

### An improved tissue clearing and high-resolution imaging method revealed intact vascular and perivascular elements in the retina

Retinal blood vessels supply nutrients and oxygen while forming the inner blood-retinal barrier (Fig. 3a), whose disruption is linked to retinal diseases.^19,38–40^ However, most retinal photodamage studies neglect vascular analysis,^17,41,42^, and traditional 2D imaging fails to capture the 3D vascular network.^43,44^ Although tissue clearing enables 3D visualization,^45,46^ its lengthy and complex protocols limit practicality.

**Fig. 4:**
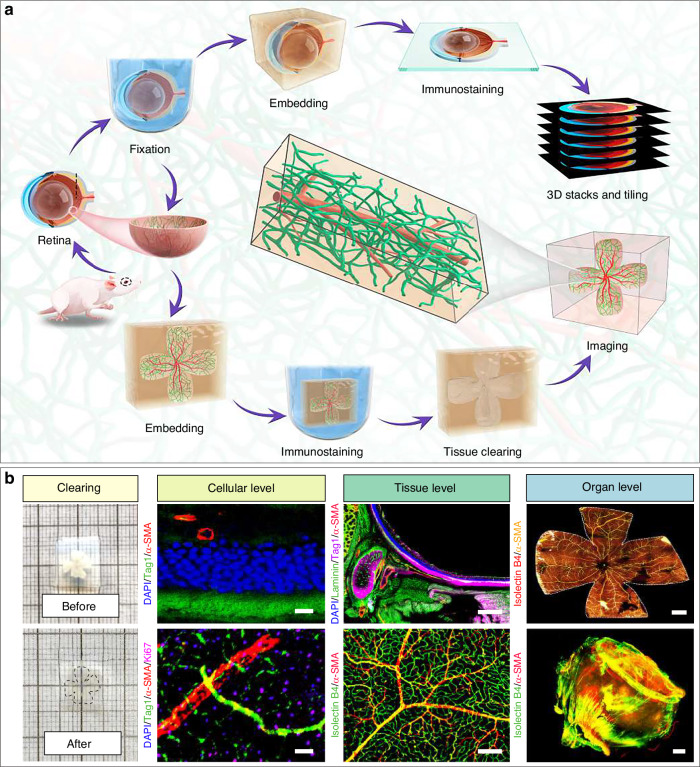
A modified multi-level high-resolution retinal tissue clearing and imaging method. **a** Schematic illustration of a modified multi-level high-resolution clearing method for whole-organ retina and eyeball imaging. **b** Representative images (left panel) of a retina prior to and post tissue clearing. Representative 3D images (center and right panels) of the retina at cellular, tissue, and organ level. Exemplar 3D images of retinas were immunostained with various antibodies as indicated, including: Laminin, α-SMA, Isolectin B4, Tag1, Ki67, and DAPI. Scale bars: (**b**) 20 μm for cellular level, 50 μm for tissue level, and 500 μm for whole-organ level

Exposure to dental lighting caused the retina to become very fragile, making it prone to rupture and deformation during the processing. To maintain the integrity of retinal tissue, a fixative solution containing acetic acid was used, which enhanced the resilience of the retina. Moreover, we embedded the samples in agarose to maintain their ideal shape. However, these steps compromised the transparency of the retina. So we modified the simple ultrafast multicolor immunolabelling and clearing (SUMIC) method previously developed by our research group^47^ to effectively apply it for imaging both the eyeball and retina (Fig. 4a and Supplementary Fig. 6a). The combination of ethyl cinnamate (ECi) and polyethylene glycol methacrylate (PEGM) rapidly altered the refractive index of the retina, rendering it transparent after only 5 min (Fig. 4a and Supplementary Fig. 6a). Briefly, retinal samples were fixed, followed by bleaching, immunostaining, isopropanol dehydration, and rapid clearing (Fig. 4a, b and Supplementary Fig. 6b). This optimized method enabled rapid and safe tissue clearing and preserved fluorescence efficiently (Fig. 4b). Light-sheet microscopy and high-resolution confocal microscopy make it possible to depict the three-dimensional vascular network of the retina and its microenvironment across multiple levels, including cellular, tissue, and whole-organ scales (Fig. 4b and Supplementary Fig. 7a).

### The dental lighting mode disrupted the inner blood-retinal barriers and decreased vascular abundance

**Fig. 5:**
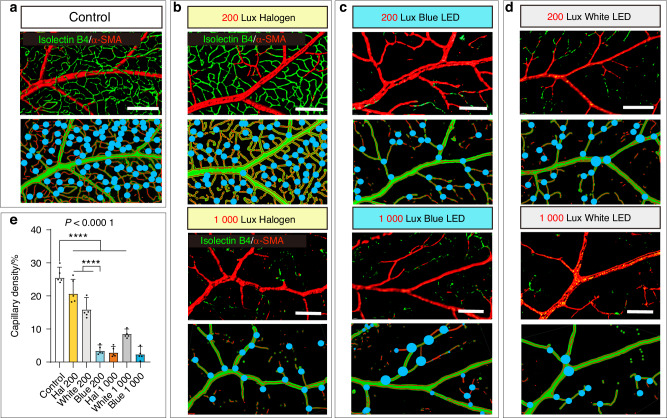
Structure changes in the inner blood-retinal barrier under the simulated dental lighting patterns. **a**–**d** Representative images of rat retinal vessels stained with α-SMA and Isolectin B4 in rats under different light patterns. Top panels show 3D reconstruction of vessels. Bottom panels show the marked vessel junction points. **e** Quantification of capillary density in different groups. Scale bars: (**a**–**d**) 90 µm. Data information: (*n* = *5*), *P* value derived from one-way ANOVA test with Student–Newman–Keuls test. ns not significant; ****P < 0.000 1. All data are Mean ± S.D

Accurate visualization of retinal vasculature is essential to assess the effects of simulated dental lighting on the inner blood-retinal barrier. Using a modified SUMIC method, we analyzed regional vessel distribution, artery and capillary density, and vessel junctions (Fig. 5a–d and Supplementary Fig. 7b). Isolectin B4 marked endothelial cells, while α-SMA staining identified artery-associated perivascular cells (Fig. 5a–d).

A healthy inner retinal barrier exhibited a healthy α-SMA^+^ perivascular cells’ attachment to the arteries, and it was surrounded by abundant capillaries (Fig. 5a). After 6 months, only the 200 Lux halogen group maintained normal vascular features comparable to controls (Fig. 5a–e), with the *P*-value of 0.219 7. However, both the blue and white LED light exposure led to a substantial reduction in capillary density under an illumination intensity of 200 Lux (Fig. 5c–e). When the light intensity increased to 1 000 Lux, the density of retinal blood vessels further decreased (*P* < 0.000 1) with all three light sources (Fig. 5b–e). Extensive non-perfused areas emerged, and complete and continuous capillary signals were not observable (Fig. 5b–d).

Moreover, vascular bifurcation parameters are crucial indicators for evaluating vascular changes in retinal structure.^48^ However, many groups have utilized a simple 2D skeletonization for bifurcation analysis and have not provided spatial information on vascular networks with three-dimensional topological structures.^49,50^ Thus, we accurately obtained capillary and junction density in a 3D structural retinal vascular network. Under the 200 Lux light intensity, the density of retinal vascular bifurcation in rats exposed to the halogen light showed no significant difference (*P* > 0.999 9) from the control group (Fig. 5b and Supplementary Fig. 7b). Conversely, the abundance of vascular bifurcation in rats exposed to blue and white LED light decreased significantly (*P* < 0.000 1) compare to those under halogen light (Fig. 5c, d and Supplementary Fig. 7b). When the light intensity increased to 1 000 Lux, the retinal vascular branching density further decreased (*P* < 0.000 1) compared to the density at 200 Lux for all three light sources (Fig. 5b–d and Supplementary Fig. 7b).

Further, vascular leakage assay was undertaken to investigate the integrity of the inner retinal barrier under various dental lighting conditions. There was minimal fluorescence leakage in both the control group and the 200 Lux halogen light group (Supplementary Fig. 7c, d). In contrast, noticeable levels of fluorescence leakage were observed in retinas exposed to other lighting conditions, with significant differences (*P* < 0.000 1) compared to the control group and the 200 Lux halogen light group (Supplementary Fig. 7c, d). These findings demonstrated the potential of dental illumination modalities to compromise the blood-retinal barrier, resulting in instances of disruption of the blood-retinal barriers.

Notably, under the simulated dental lighting patterns, there was no significant change (*P* = 0.392 5) in the retinal arteries between these groups (Supplementary Figs. 8 and 9a). Vascular loss primarily occurred in capillaries (Fig. 5b–d). In order to further analyze the perivascular cells, we focused our attention on the blue light LED groups with the most severe vascular network disruption. We applied α-SMA to label vascular smooth muscle cells, NG2 to mark perivascular cells, TAGLN to identify vascular wall cells, and GFAP to label astrocytes (Supplementary Fig. 8). Compared to the control group, the 200 and 1 000 Lux blue LED groups exhibited no alteration in α-SMA^+^ vascular smooth muscle cells and TAGLN^+^ vascular wall cells (Supplementary Figs. 8 and 9c), while concurrently displaying co-localization (Supplementary Fig. 9b). However, there was a reduction in NG2^+^ perivascular cells and GFAP^+^ astrocytes after blue LED lighting exposure (Supplementary Figs. 8 and 9c). Retinal capillaries are simple endothelial tubular structures surrounded by a single layer of basement membrane and occasional pericytes.^51^ Furthermore, our previous study demonstrated that pericytes adjacent to capillaries are susceptible to external environmental stress, leading to the differentiation of pericytes to inflammatory fibroblasts.^30^ The multiple layers of pericytes attached to arteries provide robust protective effects on arterial structures.^52,53^ Therefore, the retinal arteries can resist chronic light damage from the dental environment lighting.

Most simulated dental lighting patterns led to reduced retinal capillary structure, compromising the inner blood-retinal barrier. Capillaries were mainly affected, with minimal arterial changes. As with the outer barrier, the 200 Lux halogen group showed negligible impact, and damage severity correlated positively with light intensity.

### Retina pathology and inflammatory cell recruitment under the simulated dental lighting patterns

**Fig. 6:**
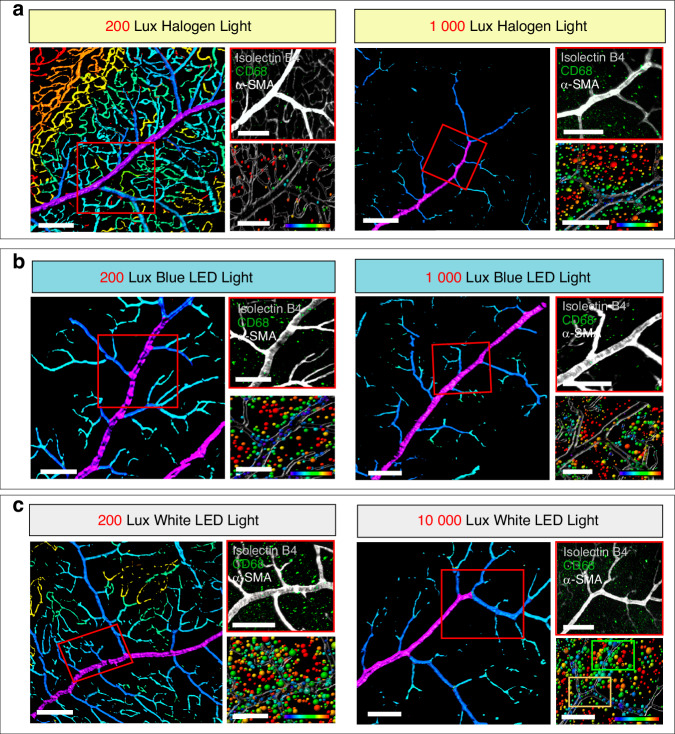
Inflammatory response in the retina under the simulated dental lighting patterns. **a**–**c** The representative 3D images (left panels) show vascular branch level rendering of retinal vessels. Vessels were marked according to their branching grade, ranging in color from purple to red. The top right panels show representative images of rat retina under the simulated dental lighting patterns, stained with CD68, α-SMA (white), and Isolectin B4 (gray). The bottom right display the 3D rendering condition, with transparent α-SMA^+^ and Isolectin B4^+^ surface rendering and colorful CD68^+^ spot rendering. The shortest distance from the spot to the surface and the cumulative number of spots at different distances were calculated. Color bars: The color of spots varies from purple to red according to the calculated distance. Scale bars: (**a**–**c**) 50 µm

It is well established that light sources with extreme color temperature or excessive light intensity can lead to oxidative stress and inflammation in the retina.^54^ Moreover, inflammation can damage the vascular endothelium,^55,56^ yet the impact of chronic photodamage-induced inflammation on retinal vasculature remains unclear. We systematically studied inner blood-retinal barrier pathology to better understand the connection between reduced intraretinal blood vessel abundance and inflammation caused by chronic dental lighting exposure. It has been demonstrated that the severity of tissue pathology increases as the branching of the vessels decreases.^57^ The vascular branch level was investigated to assess the extent of retinal vascular pathology. Through fractal analysis, blood vessels were classified and color-coded based on their respective branching level, ranging from primary arteries to small capillaries (Fig. 6a and Supplementary Fig. 9d). Under the 200 Lux light intensity, the halogen light group exhibited the highest number of retinal blood vessel branching levels, reaching up to 7 levels (Fig. 6a–c and Supplementary Fig. 9e). In contrast, the blue LED group exhibited the lowest branching levels under 200-Lux intensity, with only 3 level of vessel classification (Fig. 6b and Supplementary Fig. 9e). Under the light intensity of 1 000 Lux, all of the groups showed a remarkable reduction in retinal blood vessel branching (Fig. 6a–c and Supplementary Fig. 9e). Specifically, both the white LED and halogen light groups displayed a decrease to 3 level of vessel branching, while the blue LED group showed a further decline to only 2 level (Fig. 6a–c and Supplementary Fig. 9e).

Macrophages, as innate immune cells, play a complex role in vascular injury regulation.^58^ We investigated CD68^+^ macrophages and retinal vasculature under simulated dental working conditions. Surface rendering of α-SMA/Isolectin B4-labeled vessels and spot rendering of CD68^+^ cells revealed a curvilinear distribution of macrophage accumulation relative to vessel distance (Fig. 6a–c), with recruitment declining as distance increased. At 200 Lux, the halogen group showed minimal perivascular macrophages (Fig. 6a and Supplementary Fig. 9f), while blue LED had the highest recruitment (Fig. 6b and Supplementary Fig. 9f). At 1 000 Lux, both blue and white LEDs recruited more CD68^+^ cells than halogen (Fig. 6b, c and Supplementary Fig. 9f), with consistently higher accumulation at equal distances (Supplementary Fig. 9f). Further analysis using CD86 (M1) and CD206 (M2) markers showed increased M1/M2 macrophages in all groups (Supplementary Fig. 9g and 9h). In comparison with other groups, the 200 Lux halogen light resulted in a significantly least M1 increase (*P* < 0.000 1), with the largest M1 increase in 1 000 Lux blue LED (Supplementary Fig. 9g, h). Post-illumination, M1 macrophages localized near superficial retinal vessels, while M2 macrophages clustered in ONL/INL cells (Supplementary Fig. 9h).

Overall, the simulated dental lighting environment led to the accumulation of inflammatory cells and damage to inner blood-retinal barrier. Notably, compared to halogen light sources, both white and blue light LED lighting induced stronger inflammatory responses and elicited more pronounced vascular pathology.

### Disruption of homeostasis and vision-related functional structures within the retinal barrier under the simulated dental lighting patterns

A distinctive feature of retinal circulation is the absence of autonomous neural control, primarily regulated by glial cells and nerve fibers surrounding the blood vessels.^59^ Moreover, retinal circulation possesses unique vascular control mechanisms that are sensitive to changes in the surrounding matrix.^60^ Therefore, we subsequently investigated the correlation between changes in retinal blood vessels and modifications in retinal vision-related functional structures under simulated dental lighting modes.

**Fig. 7:**
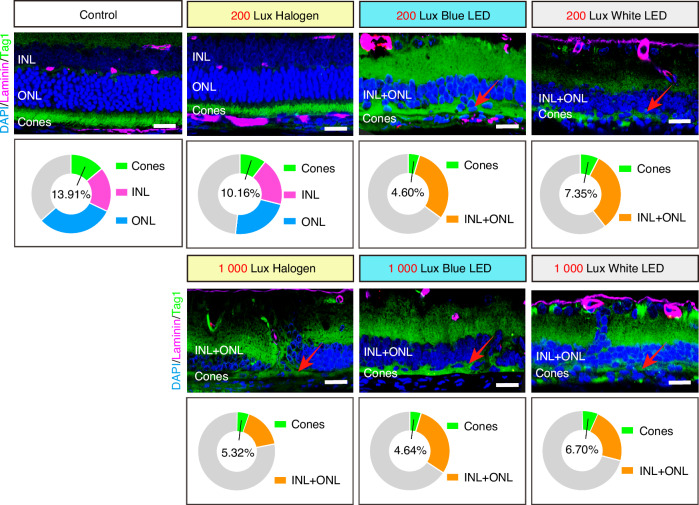
Effects of the simulated dental lighting patterns on the cones. Representative images of cones, ONL, and INL stained with Tag1, Laminin and DAPI. Green segment represents the thickness ratio of cones of retina in pie chart. ONL, outer nuclear layer; INL, inner nuclear layer. Scale bars: 20 µm

Cones and bipolar cells in the retina (Fig. 7) play a key role in transmitting light signals and discerning colors. Additionally, they are important in maintaining the retinal microenvironment’s stability and energy transmission.^51,61^ Here, Tag1 was used as a marker for cones, and Laminin was utilized to label the extracellular matrix. Under 200 Lux light intensity, the retinal cell structure in the halogen light group appeared similar to the control group (Fig. 7). In the control and 200 Lux halogen groups, the cone photoreceptor layer retained its integrity and exhibited a clustered arrangement (Fig. 7). The proportion of the cone photoreceptor layer thickness in the 200 Lux halogen group was the highest among all the lighting groups, with an average of 10.16%, and the INL and ONL showed distinct layering (Fig. 7). The cone photoreceptor layers of the retinal tissue were affected to varying degrees under other simulated dental lighting modes, and the complete arrangement of cones was almost absent, resulting in a loss of clustered arrangements (Fig. 7). The INL and ONL in these groups exhibited structural disruption and reduced thickness (Fig. 7). Under 1 000 Lux light intensity, all the three groups demonstrated a further decline in the retinal cone layer thickness, with proportions not exceeding 7% within the retina (Fig. 7).

**Fig. 8:**
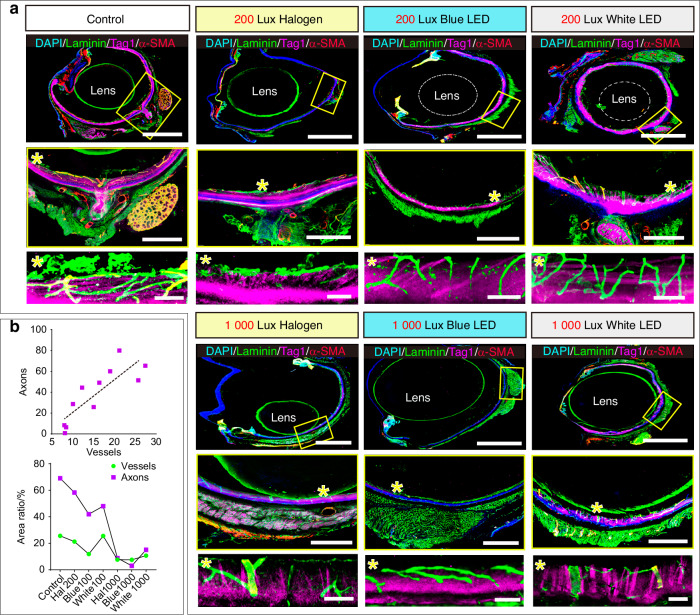
Effects of the simulated dental lighting patterns on the retinal vision-related functional structures. **a** Representative images of whole eyeball stained with α-SMA, Tag1, Laminin and DAPI. Higher magnification insets (right panels) show the regions of fundus oculi. Asterisks indicate higher magnifications of indicated regions. Hashtags show 3D reconstruction of vasculature and axons. **b** Vessel-axon correlation and quantitative analysis. The upper shows the correlation analysis between the area of vessels and axons in different groups. The lower displays the quantification of vessels and axon Scale bars: (**a**) 500 µm for tile scans, 200 µm for insets (yellow boxes), 50 µm for insets (*). Data information: (*n* = *5*), statistics were analyzed by linear regression analysis. The line is fit line

Retinal ganglion cells (RGC), a class of neuronal axons within the retina (Fig. 7), are vital visual-related structures responsible for transmitting visual information from the retina to the brain through their axons.^62^ Consequently, we analyzed the impact of changes in the blood-retinal barrier on the axons of retinal ganglion cells under the simulated dental lighting patterns. Tag1 is expressed by all sensory axons, including retinal RGC axons.^63^ We observed a linear relationship between the Tag1^+^ axons in the superficial retina and the blood vessels (Fig. 8a, b). As the density of blood vessels decreased, the density of axons also decreased (Fig. 8a, b and Supplementary Fig. 10a, b). In the control, 200 Lux halogen light and 200 Lux white LED light groups, there was a close association and mutual wrapping between retinal blood vessels and axons (Fig. 8a). However, in the 200 Lux blue LED light group, the axon-vessel interface was weakened (Fig. 8a and Supplementary Fig. 10c, d). Under 1 000 Lux light intensity, sensory axons within the retina further decreased compared to 200 Lux conditions (Fig. 8a and Supplementary Fig. 10b). Additionally, the mutual wrapping between blood vessels and axons was almost absent for blue LED light (Fig. 8a).

The above experiments showed that the inner and outer retinal barriers were impaired under the simulated dental lighting patterns, leading to further abnormalities in vision-related functional photosensitive structures and neural transmission. Notably, the blue LED light significantly damaged retinal structures at both 200 Lux and 1 000 Lux light intensities.

### Imbalance in retinal energy metabolism and immune response caused by the simulated dental lighting patterns

**Fig. 9:**
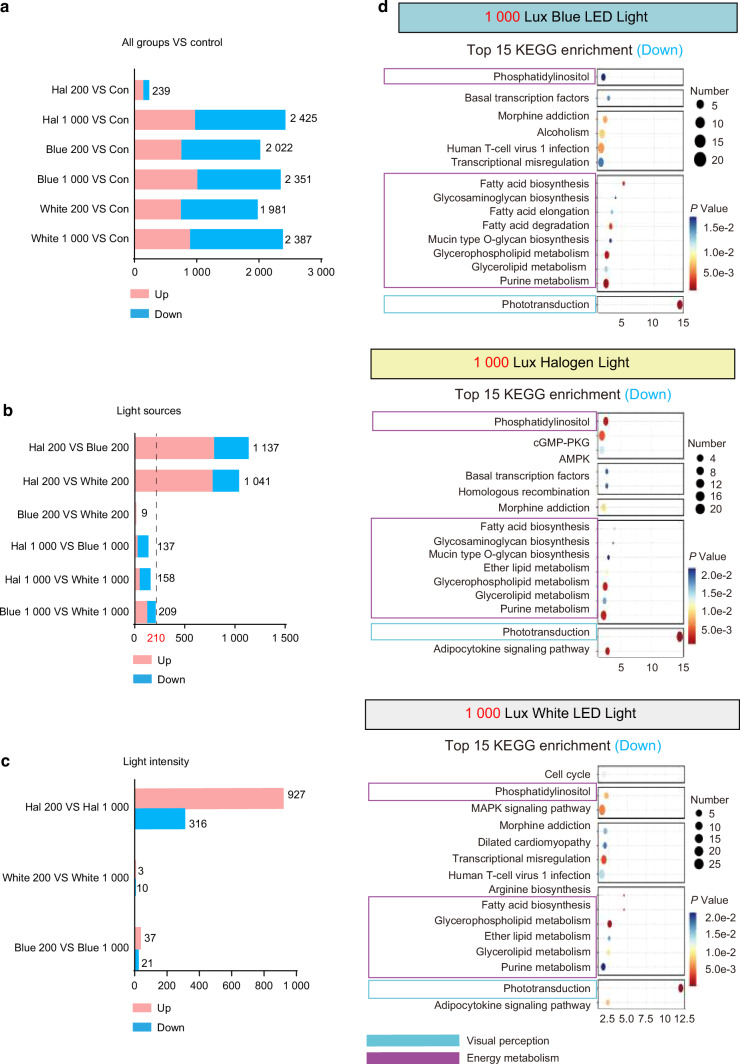
Gene analysis associated with retinal damage under the simulated dental lighting patterns. **a** Differentially expressed genes (DEGs) across light source groups vs. control. Pink: up-regulated; blue: down-regulated. Numbers indicate total DEGs. **b, c** DEGs between light sources and intensities at the same intensity. Pink: up-regulated; blue: down-regulated. Numbers indicate total DEGs. **d** Top 15 KEGG terms for down-regulated and down-regulated pathways (1 000 Lux vs. control). Bubble size = gene count; color = *P* value (blue to red)

We conducted gene analyses of retinas after 6 months of simulated light exposure to further investigate the potential factors contributing to retinal damage caused by the simulated dental lighting patterns. First, we employed sample clustering analysis to explore the data. The results revealed a robust inter-sample correlation among the samples (Supplementary Fig. 11a). Compared to the control group, all the lighting groups showed differentially expressed genes with the number 239-2 419 (Fig. 9a and Supplementary Fig. 11b). A comparison of the three types of oral light sources showed that at 200 Lux light intensity, the number of differentially expressed genes between the white LED light and the blue LED light groups was extremely small (9 genes, Fig. 9b). However, both the white and blue LED light groups exhibited a substantial number of differentially expressed genes compared to the halogen group (1 041-1 137 genes, Fig. 9b). Nevertheless, at 1 000 Lux intensity, the three groups of light sources exhibited very little variations, with <210 differentially expressed genes between each two groups (Fig. 9b). Moreover, under the same light source including the white and blue LED light sources, there were very few differentially expressed genes (<60) across different light intensities (Fig. 9c). Therefore, we observed that at low light intensities, the nature of the light source was a crucial factor contributing to retinal damage. Conversely, at high light intensities, the distinctions in light source properties were masked, leading to similar retinal damage.

**Fig. 10:**
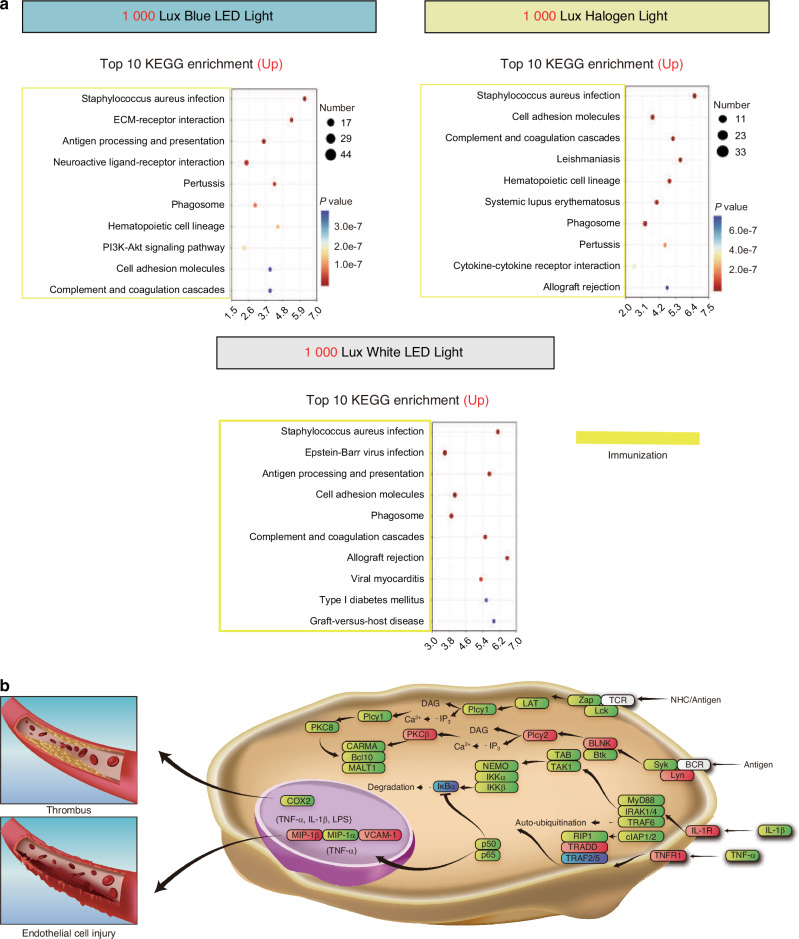
KEGG analysis and pathways associated with retinal damage under the simulated dental lighting patterns. **a** Top 10 KEGG terms for up-regulated pathways (1 000 Lux vs. control). The horizontal axis in the figure is the enrichment score. Entries with larger bubbles contain more differential protein-coding genes, and the color of the bubbles changes from blue to red according to *P*-value. **b** Proposed mechanism of retinal vascular disruption under dental lighting

We conducted the Kyoto Encyclopedia of Genes and Genomes (KEGG) and Gene Ontology (GO) enrichment analyses to gain deeper insights into the patterns of retinal damage under various light exposure conditions. The analyses showed that diseased changes primarily occurred in three aspects: visual perception system, energy metabolism, and immune response (Fig. 9d and Supplementary Fig. 11c, d). Specifically, after 6 months of exposure to simulated dental lighting patterns, the KEGG results showed that, compared to the control group, the expression of molecules related to visual perception was downregulated in all the lighting groups except for the 200 Lux halogen group (Fig. 9d and Supplementary Fig. 11c). This downregulation primarily involved processes related to phototransduction (Fig. 9d and Supplementary Fig. 11c). Furthermore, substantial downregulation of molecules associated with energy metabolism was observed in these groups, including carbohydrate metabolism and fatty acid synthesis (Fig. 9d and Supplementary Fig. 11c). Conversely, immune response processes such as cytokine interactions and phagocytosis showed significant upregulation under the simulated dental lighting patterns (Fig. 10a and Supplementary Fig. 11c). Additionally, the GO analysis also revealed upregulated inflammatory responses within the retina under simulated dental lighting patterns (Supplementary Fig. 11d).

**Fig. 11:**
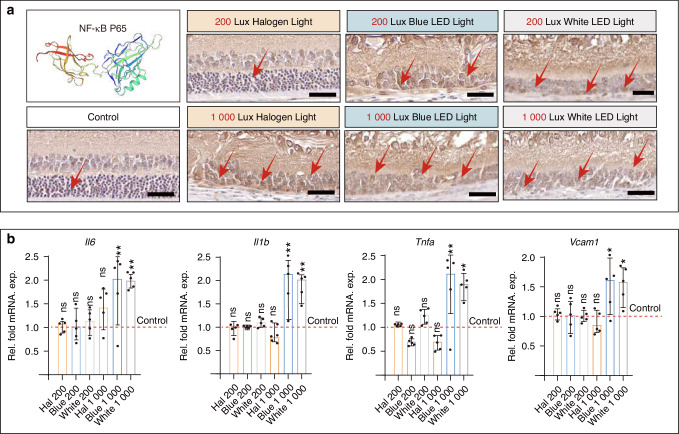
Retinal inflammatory response and change on energy metabolism under simulated dental lighting environments. **a** Retinal immunohistochemistry for NF-κB p65. Red arrows highlight antibody reactivity. **b** The histograms depict the variance in protein expression levels of IL-6, IL-1β, TNF-α, and VCAM-1 between individual groups and the corresponding control group. Scale bars: (**a**) 50 μm. Data information: (*n* = *5*), *P* value derived from one-way ANOVA test with Student-Newman-Keuls test. ns: not significant; *P < 0.05, **P < 0.01. All data are Mean ± S.D

Based on the KEGG analysis, the NF-κB signaling pathway was activated as a major inflammatory-related pathway (Fig. 10b). NF-κB p65 was used to validate these changes. Specifically, under conditions of 1 000 Lux illumination, there was a pronounced upregulation of NF-κB p65 expression within the retina (Fig. 11a). Moreover, immunohistochemical staining was performed using three inflammatory markers, TNF-α, CD68, and IL-1β (Fig. 11b and Supplementary Fig. 12). The results revealed that in the control group and the 200 Lux halogen light group, there was minimal presence of inflammation (Fig. 11b and Supplementary Fig. 12). However, under 1 000 Lux light exposure conditions, there was a significant increase in inflammation, with inflammatory regions primarily localized within ONL and INL cells (Fig. 11b and Supplementary Fig. 12). Furthermore, we conducted a pathway analysis, focusing on the representative 1 000 Lux blue LED group versus the control group. We detected an upregulation of extranuclear proteins such as IL-1R, TNF-R1, BLNK, PKC8, and intranuclear proteins like MIP-1β and MIP-2 (Fig. 10b). These gene alterations are likely to result in an increased production of inflammatory-related cytokines such as IL-6, IL-1β, TNF-α, and VCAM-1, consistent with the qPCR results (Fig. 11b).

Previous studies have shown that in tissue injury states, the regression of blood vessels is positively correlated with energy metabolism processes such as glucose and fatty acid metabolism.^64,65^ To validate the result of reduced energy metabolism based on GO analysis, we conducted in vivo experiments. We applied immunohistochemical staining with PI3K p85 (Supplementary Fig. 12). PI3K p85 exhibited low expression in the retinas of the control group and the 200 Lux halogen light group, predominantly in the nuclear layer of the retina (Supplementary Fig. 12). As the light intensity increases from 200 Lux to 1 000 Lux, there is a significant increase in the expression of PI3K p85, predominantly located in the inner and outer nuclear layers (Supplementary Fig. 12). Elevated expression of PI3K p85 inhibits the AMP-activated protein kinase (AMPK) pathway,^66^ indicating a weakened cellular energy state.

Under the simulated dental lighting patterns, there was a notable regression of capillaries in the rat retina, which affected blood perfusion, resulting in abnormal retinal energy metabolism. Simultaneously, chronic light damage induced macrophage recruitment around blood vessels and the activation of the Nf-κB pathway, further contributing to blood-retinal barriers impairment and degeneration of vision-related functional cells and sensory axons. Based on these findings, it is shown that the main potential causes of retinal homeostasis impairment induced by chronic oral light damage are imbalances in energy metabolism and immune responses. The *Blnk* and *Ccl4* genes within the Nf-κB pathway serve as a potential regulatory target.

## Discussion

Dentists often work in environments with multiple harmful light sources for extended periods, resulting in a higher risk of ophthalmic diseases than other professions. Despite the availability of relevant epidemiological research, there is still a lack of animal models that simulate the dental work environment lighting and fundamental experimental data concerning the impact of chronic photodamage on the retina. This research investigated the higher susceptibility of dentists to visual function abnormalities compared to non-dentists through an epidemiological survey. We implemented a large-scale 3D spatial comparison of retinal vasculature affected by chronic photodamage under multiple dental lighting conditions. This comprehensive analysis of retinal damage emphasizes the importance of vascular damage as a pathological alteration, delving into the effects of chronic photodamage from dental light sources on retinal homeostasis and visual-related functional structures. Moreover, retinal vasculature undergoes dynamic changes to fulfill its energy requirements,^51^ which was validated by our experiments since the reduction in the retinal vascular network is accompanied by a decrease in retinal energy metabolism. Additionally, the activation of the NF-κB pathway appears to be closely linked to the inflammatory damage of the retinal vasculature.

In this study, we explored the retinal impact of chronic exposure to common dental light sources, focusing on the long-term effects. While light exposure has been extensively studied in the context of acute retinal injury, few studies have examined the cumulative effects of extended light exposure, especially in occupational settings like dentistry. Therefore, we performed a longitudinal assessment of retinal changes at 1-month, 3-month, and 6-month intervals. We found that without compromising animal welfare, significant intergroup differences could be observed at 6 months, highlighting the cumulative nature of retinal damage. Our imaging dataset revealed that the loss of vascular abundance, accompanied by the recruitment of inflammatory cells, is a key characteristic of chronic retinal photodamage under the dental work environment lighting. This phenomenon has been relatively infrequently observed in previous studies on photodamage.^9^ Considering the retinal microenvironment, retinal blood vessels are highly sensitive to changes in homeostasis. Light exposure leads to elevated reactive oxygen species levels within the retinal environment, disrupting mitochondrial function in retinal vascular endothelial cells and leading to endothelial cell apoptosis.^67,68^ Moreover, light exposure can impair energy metabolism within the retina. Due to the unique spatiotemporal heterogeneity of retinal capillaries, the retina can restructure its capillary network according to metabolic demands.^51^ Considering inflammatory factors, leukocytes are indirectly activated through cytokine release when retinal metabolism is dysregulated. Once metabolic changes become pathogenic, physiological alterations occur in both endothelial cells and blood leukocytes, promoting abnormal leukocyte-endothelial adhesion, leading to vascular occlusion and subsequent vascular damage in the retina.^57^ Additionally, inflammatory cascades around retinal blood vessels damage the vascular-neuronal units, impairing both retinal vascular structure and function.^69^ As individuals age, the retina may undergo various changes, including inflammation within the retina, sclerosis, and narrowing of retinal vessels leading to impaired blood flow, and irregular alterations in the retinal pigment epithelium. Interestingly, these changes exhibit similarities to alterations observed in retinal tissues under dental lighting patterns, such as characterized primarily in the accumulation of Lipofusin autofluorescence and the infiltration of inflammatory cells. Thus, these lighting patterns might potentially accelerate the aging process of the retina. More extensive molecular research is necessary to elucidate the mechanisms underlying retinal pathologies resulting from aging and photodamage, facilitating the development of targeted treatments for associated ocular diseases.

While we have thoroughly investigated the chronic photodamage to the retina under simulated dental working conditions, there are still certain limitations. First, we must consider the inherent differences between the human retina and the rat retina. The most significant distinction lies in the number and density of photoreceptors.^70^ The species and anatomical variations may contribute to different responses to photodamage. Thus, rigorous longitudinal studies are required when investigating chronic photodamage in humans. But the observed changes were also consistent with the molecular and cellular mechanisms implicated in light induced retinal damage, including inflammation, and apoptosis, which have been shown to be highly conserved across species. Importantly, while structural differences exist between rodent and human retinas, such as the absence of a macula in rodents and a higher rod-to-cone ratio, the fundamental organization of retinal layers and the key cellular responses to phototoxicity are largely consistent. Rats are widely used in light-induced retinal degeneration studies because their retinal structure is well understood, they respond consistently to light, and they tolerate long term exposure experiments well.^9,71^ Several studies have demonstrated that light-induced oxidative stress, RPE dysfunction, and photoreceptor loss observed in rodent models share common pathological pathways with human conditions such as age-related macular degeneration (AMD).^72–74^ Therefore, while interspecies differences should be considered when interpreting the results, the use of rats in our study provides a potentially reliable and translationally relevant model for investigating cumulative retinal responses to occupational lighting exposures. Secondly, more precise and non-invasive observation methods should be developed to better assess the complex vascular network changes resulting from chronic photodamage. Moreover, further investigation is required to explore the specific mechanism of dental environment lighting-induced damage to the blood-retinal barrier, particularly focusing on the regulatory targets within the Nf-κB pathway. Targeted drug development for chronic retinal photodamage should also be pursued.

In brief, based on our research findings, dental lighting was found to disrupted both inner and outer blood-retinal barriers, reduced retinal blood vessels, and promoted perivascular macrophage recruitment. So we propose the following recommendations for dental lighting usage guidance:

1. Under good visibility conditions, it is advisable to minimize the light intensity and opt for a weaker light mode.
2. With low intensity, halogen lights have less impact on the retina and can also be considered an alternative light source for illumination.
3. Blue light causes the most significant damage. Dentists using blue light curing lights must not directly stare at the light and should adopt protective measures.
4. White LED light is the most commonly used light source for dental illumination. However, we found that it contains blue light components, which can damage the retinal barriers both inside and outside the eye. Therefore, modifications can be made in the manufacturing process of dental illumination light sources to ensure adequate brightness and avoid using blue light and other short-wave components.

## Methods

### Human data collection

Data pertaining to the participants’ visual health were extracted from the West China Hospital physical examination records. Vision-related health issues were identified based on the International Classification of Diseases, Tenth Revision (ICD-10) codes included H35 (retinal disorders), H52 (visual disturbance) and H53 (visual function anomalies). The prevalence of vision-related health issues was then calculated for both dentists and non-dentists.

### Animals

Sprague-Dawley rats were obtained from Chengdu Dashuo Bio-Technology Co., Ltd. (China). Rat at ages 8 weeks, male. Rats were maintained on a 12 h/12 h light-dark cycle at 22 °C with luminance below 250 Lux for 21 days before light-exposure experiments. All procedures were performed in accordance with the Association for Research in Vision and Ophthalmology statement for the use of animals in Ophthalmic and Vision Research and the Research Ethics Committee of West China Hospital of Stomatology, Sichuan University (WCHSIRB-D-2022-291).

### Light sources

Three types of light devices (Philips Lighting, China) were used, each with two intensities, creating six different light modes. Devices included cold-white LED (pure white 6 300 K), blue LED ( > 9 999 K), and Halogen (3 000 K). Light intensity was set at either 1 000 Lux or 200 Lux. At 200 Lux, the spectral irradiance (mW/m²/nm) was 2.1 for halogen, 16.8 for blue LED, 4.8 (blue light part), and 3.8 (yellow light part) for white LED. At 1 000 lux, values increased to 20.3 for halogen, 480.3 for blue LED, 24.1 (blue light part), and 12.2 (blue light part) for white LED. Devices were positioned above six transparent cages, spaced for air circulation, and constant temperature was maintained at 21 °C. Light sources were positioned vertically above each cage, each equipped with the same 10 bulbs and surrounded by diffuser panels to ensure uniform light distribution.

### Establishment of photodamage models for simulating dental lighting patterns

SD rats were conditioned in a cyclic light/dark environment (250 Lux, 12 h/12 h) for 21 days. 140 rats were randomly allocated into 7 groups using random number method, without blinding, with each group consisting of 20 rats. To mimic the working conditions of dentists, rats were only placed in the light-exposure chamber during the 8-h daily exposure window, 5 days per week, for 6 months. During non-exposure time, rats were returned to cages with standard housing conditions.

### Optical coherence tomography (OCT) and fundus autofluorescence (FAF)

Rats were anesthetized with Alpha Chloralose (40 mg/kg i.v. bolus, followed by 40–50 mg/kg/h) and examined with our OCT and FAF system. OCT images were taken with Heidelberg Spectralis OCT (Heidelberg OCTplus HRA + OCT, Heidelberg, Germany), with the fovea reflection as a reference. The device was set to 97 sections mode for retinal data. FAF images were obtained using short-wavelength excitation (488 nm) with a scanning laser ophthalmoscope.

### Fluorescein fundus angiography

After anesthetizing the rats, tail vein injection of 10% sodium fluorescein contrast agent (0.3 mL per 100 g) was administered. Upon venous filling, retinal images were captured at 2-min intervals, focusing on pathological areas. Imaging continued for at least 10 min or until the gradual disappearance of intravascular sodium fluorescein contrast agent was observed.

### Histology

Rats were euthanized, and organs were dissected, collected, photographed, and fixed with 10% formalin in PBS. After embedding in paraffin, samples were sliced into 5-µm sections, stained with H&E, and imaged with an upright optical microscope (DM500, Leica Co., Germany).

### Sample preparation for immunostaining

Eyeballs were dissected from SD rats and fixed in ice-cold 4% paraformaldehyde (Sigma-Aldrich, P6148) for 4 h. Samples were washed with PBS, treated with 20% sucrose (Sigma-Aldrich, V900116) and 2% polyvinylpyrrolidone (PVP) (Sigma-Aldrich, P5288) solution at 4 °C for 12 h, and embedded in an 8% gelatin solution with 20% sucrose and 2% PVP. Sections (100-µm thick) were cut with a Leica CM1950 cryostat (Leica Co., Germany) and air-dried at room temperature for 2 h, then stored at −20 °C.

### Immunostaining

Sections were thawed, air-dried, hydrated with PBS, and permeabilized with 0.3% Triton X-100 (Sigma-Aldrich, T8787). Blocking was done with 5% donkey serum (Sigma-Aldrich, T8787) in PBS at room temperature (RT). Sections were incubated with primary antibodies diluted in blocking buffer (1:150) for 4–4.5 h at RT, washed with PBS, and then incubated with Alexa Fluor–conjugated secondary antibodies (1:300) for 1–1.5 h at RT. After rinsing, sections were sealed with Fluoromount-G and counter-stained with DAPI. They were stored at 4 °C until imaging. Primary and secondary antibodies were used as listed in Supplementary Tables 2 and 3.

### Immunohistochemistry

Samples were fixed, embedded in paraffin, and sectioned. Paraffin sections were baked at 60 °C for 30–60 min, followed by section treatment. Sections were treated with 40 mL PBS, 120 μL Triton X-100, and 400 μL 30% H_2_O_2_ (Sigma-Aldrich, HX0636), followed by three PBS washes for 3 min each. Subsequently, sections were soaked in 0.01 mol/L sodium citrate buffer (Sigma-Aldrich, P4922), boiled in a microwave at high power for 4 min until boiling, and cooled naturally to room temperature. Tissues were blocked with serum from the same species as the secondary antibody at 37 °C for 30 min, and diluted primary antibody was added and incubated at 37 °C for 1–2 h. After three PBS washes for 3 min each, diluted secondary antibody was added and incubated at 37 °C for 1–2 h. PBS washes were performed three times for 3 minutes each. DAB-H_2_O_2_ (Yeasen, 36303ES01) staining was carried out for 10 minutes, followed by termination with distilled water. Hematoxylin (Yeasen, 60502ES50) staining was performed for 30 s and immersion in running water for 15 min. Dehydration was performed in ethanol (Sigma-Aldrich, AX0045) gradients (from low to high: 50%, 70%, 95%, 100%) for 2 min each. Finally, sections were made transparent in xylene for 5 min and mounted with neutral resin for observation.

### The modified tissue clearing procedure for retinal imaging

The SUMIC-based modified tissue clearing and whole-mount immunostaining technique was employed in this study. Rats were humanely euthanized, and their eyeballs were dissected. Subsequently, the eyeballs were fixed in 4% PFA for 4 h, and the cornea, sclera, lens, and choroid were delicately removed, preserving the retina. To enhance tissue resilience and minimize potential damage, the retina was treated with FAS (Servicebio, G1109) for 10 min and then embedded in a 3% agarose gel (Macklin, 9012-36-6). After a gentle wash with PBS, the samples were immersed in a buffer solution comprising 25% urea (Sigma-Aldrich, U5128), 15% glycerol (Sigma-Aldrich, G7893), and 7% Triton X-100 for 6–12 h at 4 °C. Subsequently, a wash buffer containing 2% fetal bovine serum (FBS) (Solarbio, S9020) in PBS was used for two 5-min washes to reduce non-specific binding. To prevent unspecific interactions, the samples were incubated with a blocking buffer consisting of 10% donkey serum, 10% dimethyl sulfoxide (DMSO) (Sigma-Aldrich, 472301), and 0.5% Triton X-100 at 37 °C for 20 min.

Next, the samples were exposed to primary antibodies, appropriately diluted in an antibody solution (1:150) prepared with 2% v/v donkey serum, 10% DMSO, and 0.5% Triton X-100 in PBS. The retina was allowed to incubate overnight at RT, followed by a 3-h washing step with a washing buffer (2% v/v donkey serum and 0.5% Triton X-100 in PBS) at RT. In the case of unconjugated primary antibodies, the samples were further incubated with secondary antibodies for 6 h at RT, using the same antibody solution. After the incubation with secondary antibodies, the samples underwent a 3-h washing step at 37 °C using the washing buffer. Subsequent to the immunostaining process, the samples were dehydrated by immersion in an isopropanol (Sigma-Aldrich, 190764) gradient (30%, 50%, 80%, and 100%) for 15 min each at RT. Finally, the sample was rinsed in a solution containing 80% ECi (Sigma-Aldrich, 112372) and 20% poly(ethylene glycol) methyl ether methacrylate (PEGM) (Sigma, 447943) until transparency was reached. Primary and secondary antibodies were used as listed in Supplementary Table 2 and 3.

### Imaging setup and image acquisition

Images were collected using a spinning disk confocal microscope (Andor Dragonfly 200, Oxford Instruments, England) and a light-sheet microscope (Miltenyi-LaVision Biotech UltraMicroscope II, Miltenyi Biotec, Germany). The imaging setup incorporated four laser lines (405, 488, 561, 647 nm) and six emission filters (420 nm–730 nm). Acquisition and analysis were performed using software modules that enabled measurement, multi-channel visualization, panorama, manual extended focus, image analysis, time-lapse, Z Stack, extended focus, autofocus, and auto-stitching. All imaging settings were kept consistent between samples from different groups for each channel. Z-stacks of images were processed and reconstructed in three dimensions using Imaris software (Version 9.7, Oxford Instruments, England). Further image processing and analysis were conducted with Imaris, Adobe Photoshop, and Adobe Illustrator, adhering to the Nature Communication guidelines for image processing.

### 3D rendering

Surface rendering: After defining the Region of Interest (ROI), individual vascular channels, perivascular cells, or stromal markers were reconstructed by surface segmentation. Threshold adjustments were made until satisfactory surfaces were previewed.

Spot rendering: Spots were defined and classified based on mass and intensity, and those outside tissue regions were manually deleted.

Filament rendering: Blood vessels were reconstructed using filament segmentation. Bounds were set in the connective baseline, and the threshold was adjusted to match the vessel volume.

### Quantifications of imaging datasets

Quantification of Signal Expression Analysis of vessel density, nerve density, vessel diameter, and signal colocalization was performed using Imaris (Version 9.7) and Fiji (Version 1.53q) software. To assess fundus autofluorescence area, leakage area, vessel density, and the percentage of perivascular signals, we adjusted the threshold in Fiji to delineate the signal area within the analyzed region and the total area of the retina. Signal quantification was determined by dividing the signal area by the total area of the selected region. Vessel density was calculated as the tissue volume of vessels divided by the total tissue volume of the entire organ. Arterial and vessel numbers were quantified using channels of α-SMA and Isolectin B4, respectively. Further details on the analysis of junction number and branch levels of vessels were conducted with AngioTool and Imaris. CD68 immunostaining was used to identify and quantify macrophage numbers.

### Analysis of colocalization

Colocalization analysis was conducted using the Fiji Colocalization Finder function. Upon opening the required images for analysis, the fluorescence channels were separated. The Colocalization Finder function was then utilized to assess the colocalization of the target fluorescence signals, resulting in the generation of colocalization scatter plots and quantitative data.

### Quantitative PCR

Retina samples were collected and homogenized before cDNA synthesis. Specific primers were designed using Primer Premier and synthesized by Shanghai Sangon Bioengineering Technology Service Co., Ltd. Primer sequences are listed in Supplementary Table 4. The reagents used for qPCR: The reagents used for qPCR: Molpure® Cell/Tissue Total RNA Kit (19221ES50, YEASEN Corporation, China); PrimeScript RT reagent Kit (RR047A, BaoRui Biotechnology Co., Ltd, China); TB Green TM Premix Ex TaqTM ⅡBaoRui Biotechnology Co., Ltd, China); Anhydrous Ethanol (2207181, Sichuan Xilong Scientific Co., Ltd, China).

### RNA isolation

Fresh retinal tissue samples (10 mg each) were added to 500 μl of lysis buffer and homogenized using a high-speed, low-temperature tissue grinder. The homogenized lysate was transferred to a centrifuge tube and centrifuged at 12 000 r/min for 5 min to collect the supernatant. The collected supernatant was transferred to DNA purification column A2, and centrifuged at 12 000 r/min for 2 min to retain the filtrate. To the retained filtrate, 1.6 times the volume of protein removal solution was added, and gently mixed by pipetting. The entire mixture was loaded into an RNA adsorption column and centrifuged at 12 000 r/min for 1 min, discarding the filtrate. Subsequently, 500 μL of binding solution was added to the column, followed by centrifugation at 12 000 r/min for 1 min, discarding the filtrate. The RNA adsorption column was placed back into a collection tube, 700 μL of wash buffer was added, and centrifuged at 12 000 r/min for 1 min, discarding the filtrate. The RNA adsorption column was then placed in a new centrifuge tube, and 50 to 100 μL of RNAase-free H_2_O was added to the center of the membrane. It was left at room temperature for 2 min and then centrifuged at 12 000 r/min for 1 min. The eluate was collected and stored at −80 °C. Transcriptome libraries were constructed, and sequencing and analysis were conducted by OE Biotech Co., Ltd. (Shanghai, China).

### RNA-seq data analysis

After sequencing on an Illumina Novaseq 6000 platform, raw reads were processed, mapped, and analyzed. Differential expression analysis and enrichment analyses were performed, including GO, KEGG pathway, Reactome, and WikiPathways, using R (v 3.2.0). Column, chord, and bubble plots were generated, and GSEA software was utilized for gene set enrichment analysis.

### RNA-seq data Link

https://www.ncbi.nlm.nih.gov/geo/query/acc.cgi?acc=GSE254856.

### Statistical analysis

Statistical analyses were processed with GraphPad Prism (GraphPad Software, USA, version 9.0) and IBM SPSS Statistics (IBM, USA, version 24.0). The prevalence rates were compared between dentists and non-dentists using chi-square tests. We applied logistic regression models to adjust for potential confounding factors, including age, and occupation. The results were presented as odds ratios (ORs) with 95% confidence intervals (CIs). Differences were calculated using one-way ANOVA with Student–Newman–Keuls test. Data represented the mean ± S.D. of at least three replicates, with various levels of statistical significance denoted by *P < 0.05, **P < 0.01, ***P < 0.001, and ****P < 0.000 1; “ns” denotes not significant.

## Supplementary information

Supplementary Materials

## Data Availability

Source data are provided in this paper. The remaining data are available within the Article, Supplementary Information, or Source Data file.
