# Supplementary material for: Chronic dental lighting disrupts blood-retinal barrier homeostasis via vascular and inflammatory pathways
Source: Int J Oral Sci. 2026 Feb 13;18:11. doi: 10.1038/s41368-025-00414-3 (PMC12902021; doi:10.1038/s41368-025-00414-3)
Supplement: Supplementary file 1 — Supplementary Materials [file 41368_2025_414_MOESM1_ESM.pdf]

Supplementary materials

Chronic Dental Lighting Disrupts Blood-Retinal Barrier Homeostasis via Vascular and Inflammatory Pathways

Haiyang Sun[1,†], Shuhuai Meng[2,3†], He Cai[1], Zhengyi Xu[1], Kuo Gai[1], Dan Meng[4], Yixin Shi[1], Feng Luo[1], Xibo Pei[1], Jian Wang[1], Anjali P. Kusumbe[2,3,*], Qianbing Wan[1,*] and Junyu Chen[1,*]

Supplementary Figures

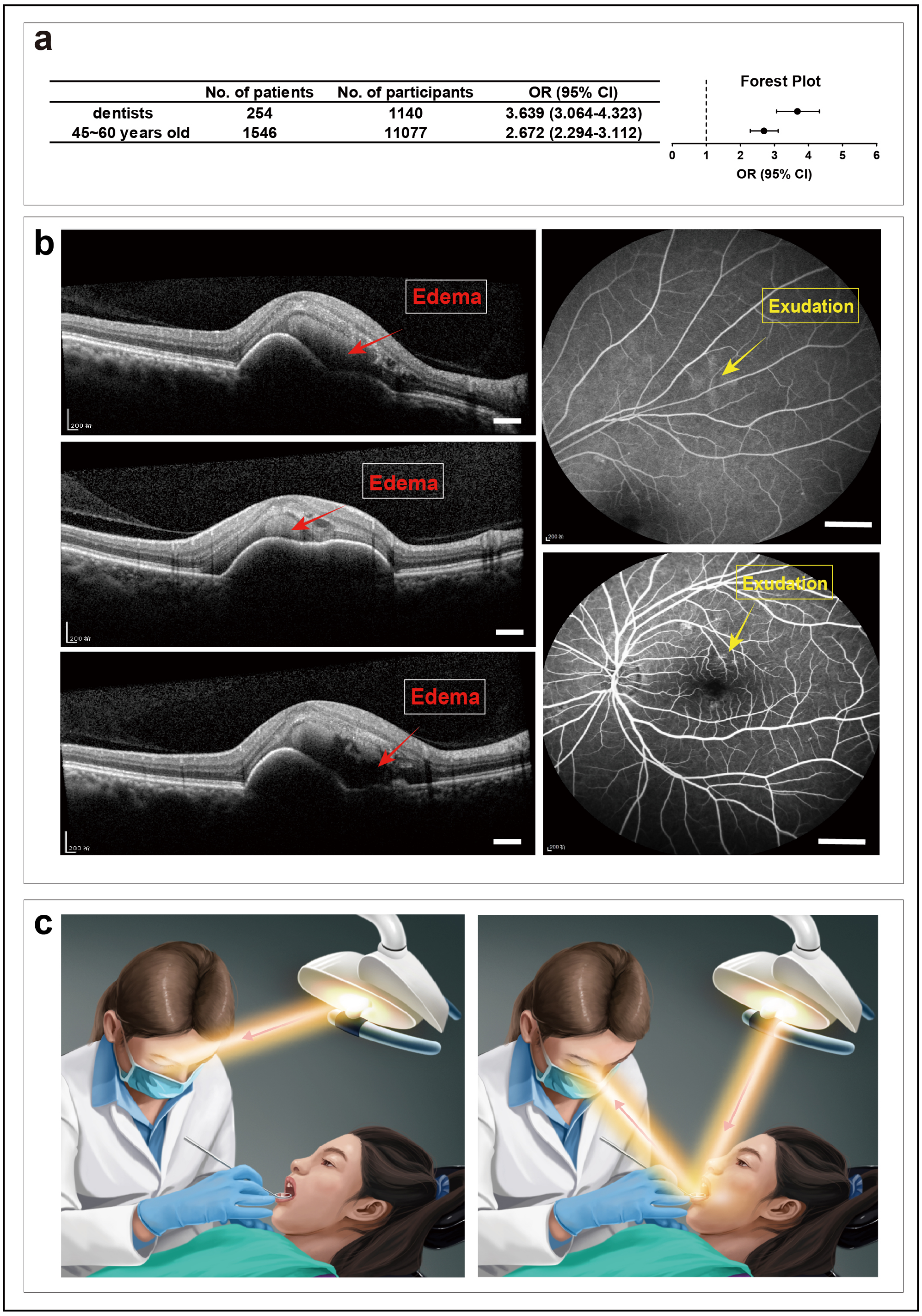

Supplementary Fig. 1 Retinal pathology and light exposure in dentists. a The three-line table on the left displays the OR values and 95% CIs from the logistic regression analysis, while the forest plot corresponding to this regression is shown on the right. **b** Left panels show representative OCT slice images displaying retinal pathology from a dentist. The areas indicated by red arrows correspond to regions of retinal edema. Right panels show representative indocyanine green angiography images displaying retinal pathology from a dentist. The areas indicated by yellow arrows correspond to regions of exudation. **c** Schematic diagram of direct light entering the eyes and schematic diagram of reflection from the oral mirror entering the eyes during the daily work of dentists. Scale bars: (b) 600 μm for left panels, 1000 μm for right panels.

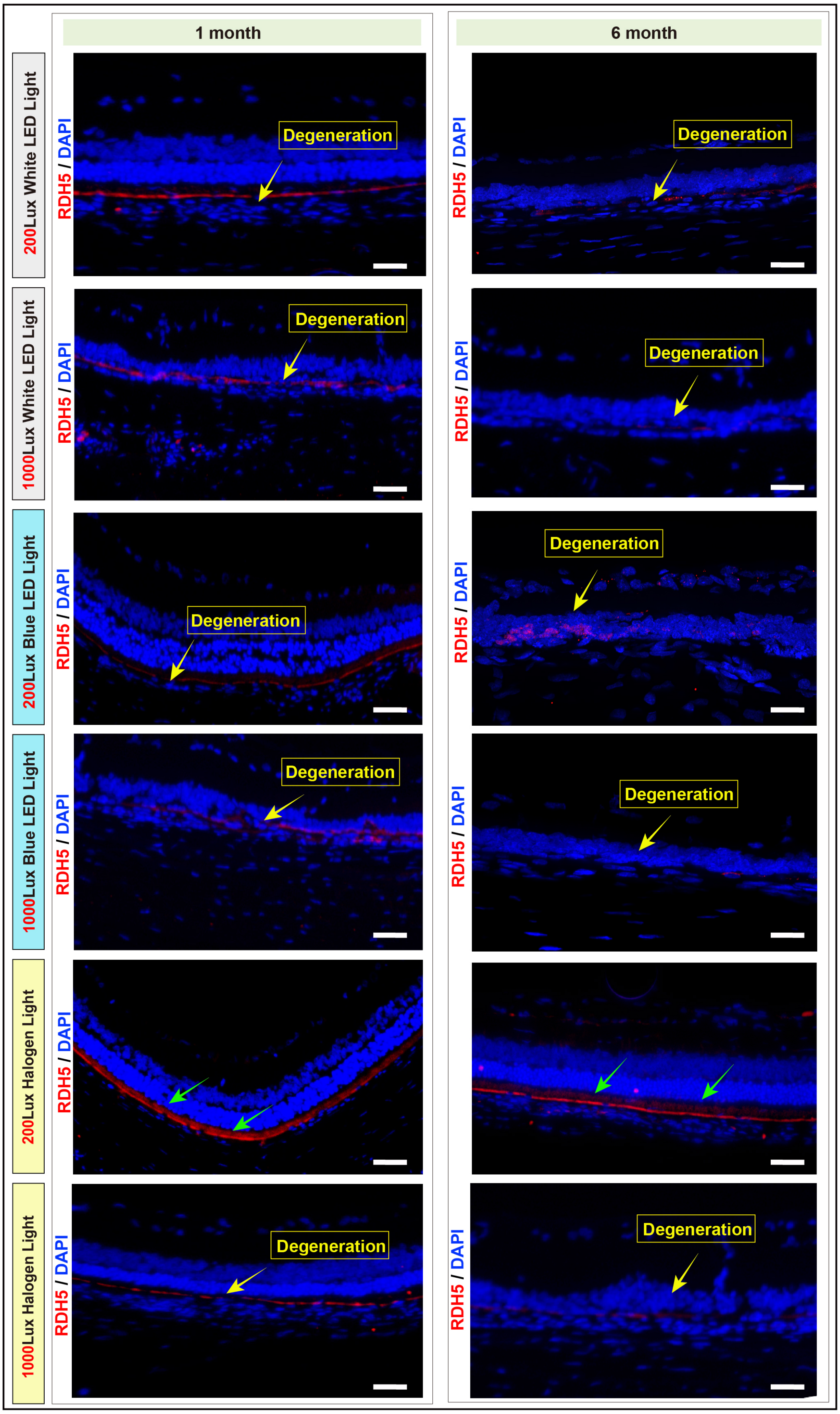

Supplementary Fig. 2 Representative immunofluorescence images at 1-month and 6-month illustrating the RPE region stained with RDH5 and DAPI. Green arrows indicate the healthy RPE layer, and yellow arrows indicate the degeneration of the RPE layer. Scale bars: 50 µm.

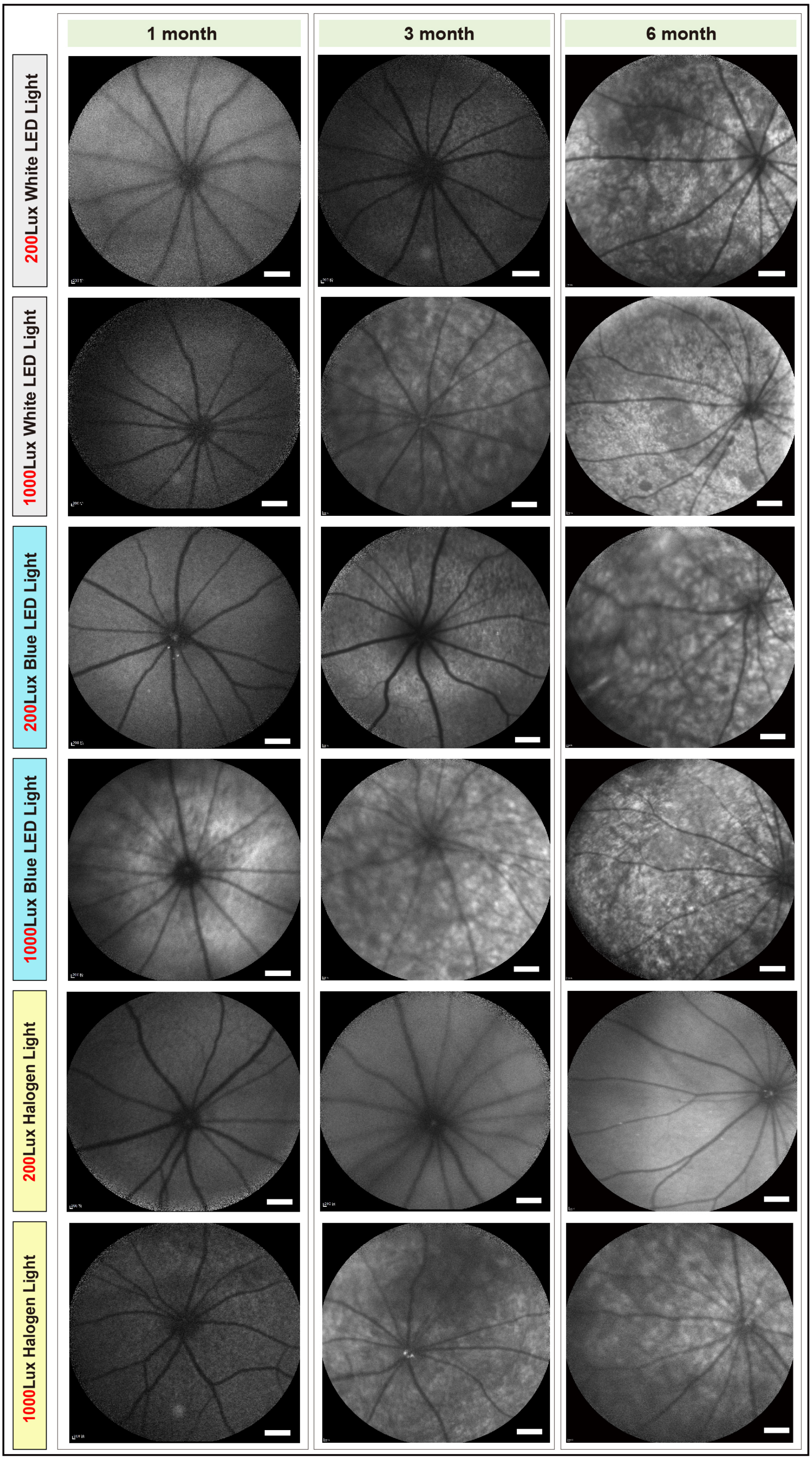

Supplementary Fig. 3 Representative images of spontaneous fluorescence from the lateral view of eyeballs in each group at the time points of 1, 3 and 6-month. Scale bars: 1000 μm.

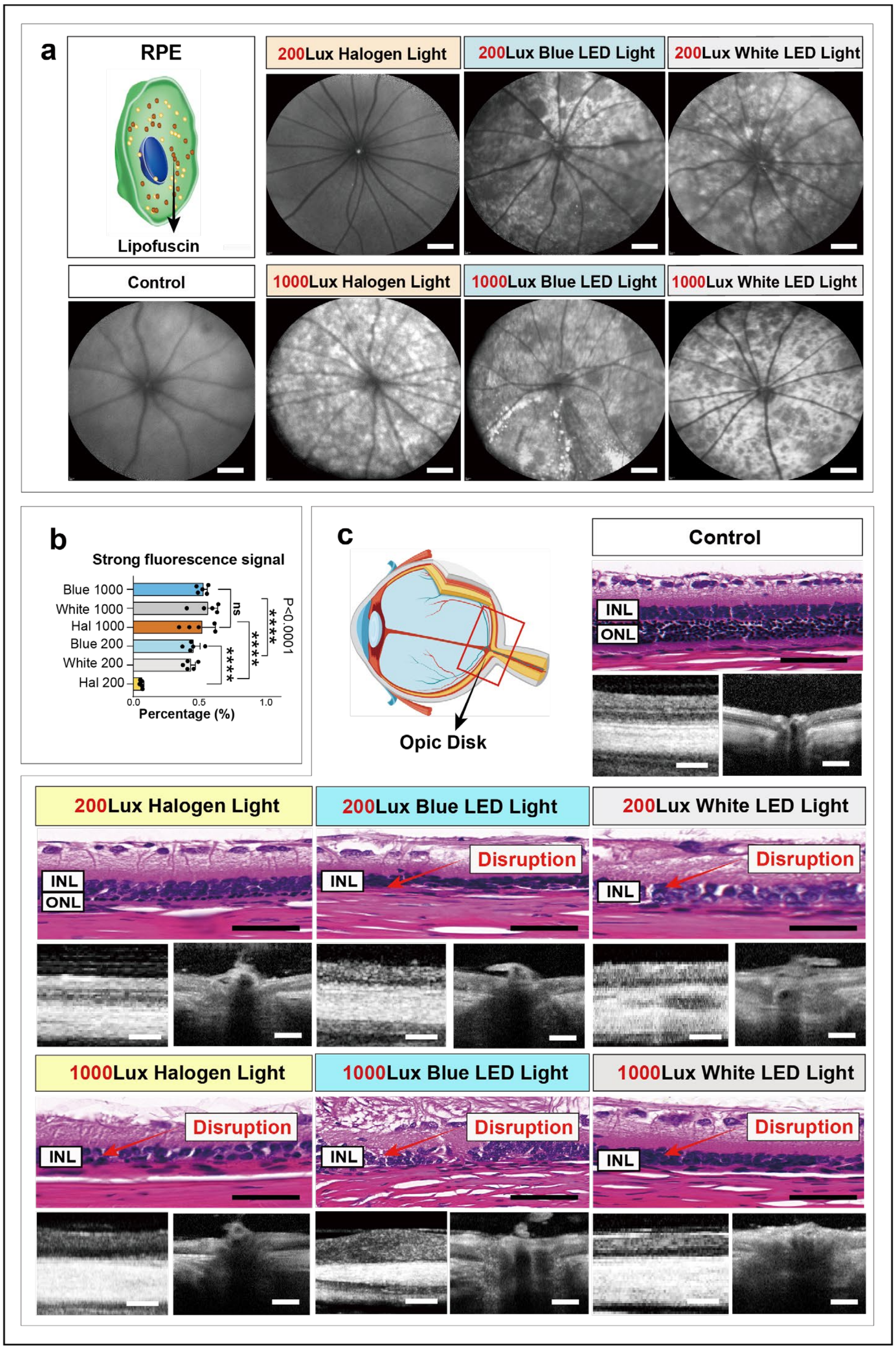

Supplementary Fig. 4 Effects of the simulated dental lighting patterns on the outer blood-retinal barrier after 6-month exposure. a The top left panel depicts a schematic representation of cumulative lipofuscin in RPE. Other panels show representative retinal images of fundus autoflourescence (FAF). **b** Quantification of the proportion of strong fluorescence signal area based on FAF images. **c** The top left panel illustrates a schematic diagram of the observation area focused on the optic disc. Other panels show representative retinal images of H&E staining (colorful) and OCT (monochrome). Red arrows indicate locations where disruption occurs in the ONL and INL. ONL, outer nuclear layer; INL, Innerer nuclear layer; RPE, retinal pigment epithelium. Scale bars: (b) 1000 μm; (c) 50 μm for HE staining, and 200 μm for OCT.

Data information: (*n=5*), P value derived from one-way ANOVA test with Student-Newman-Keuls test. ns: not significant; ****P < 0.0001. All data are Mean ± S.D.

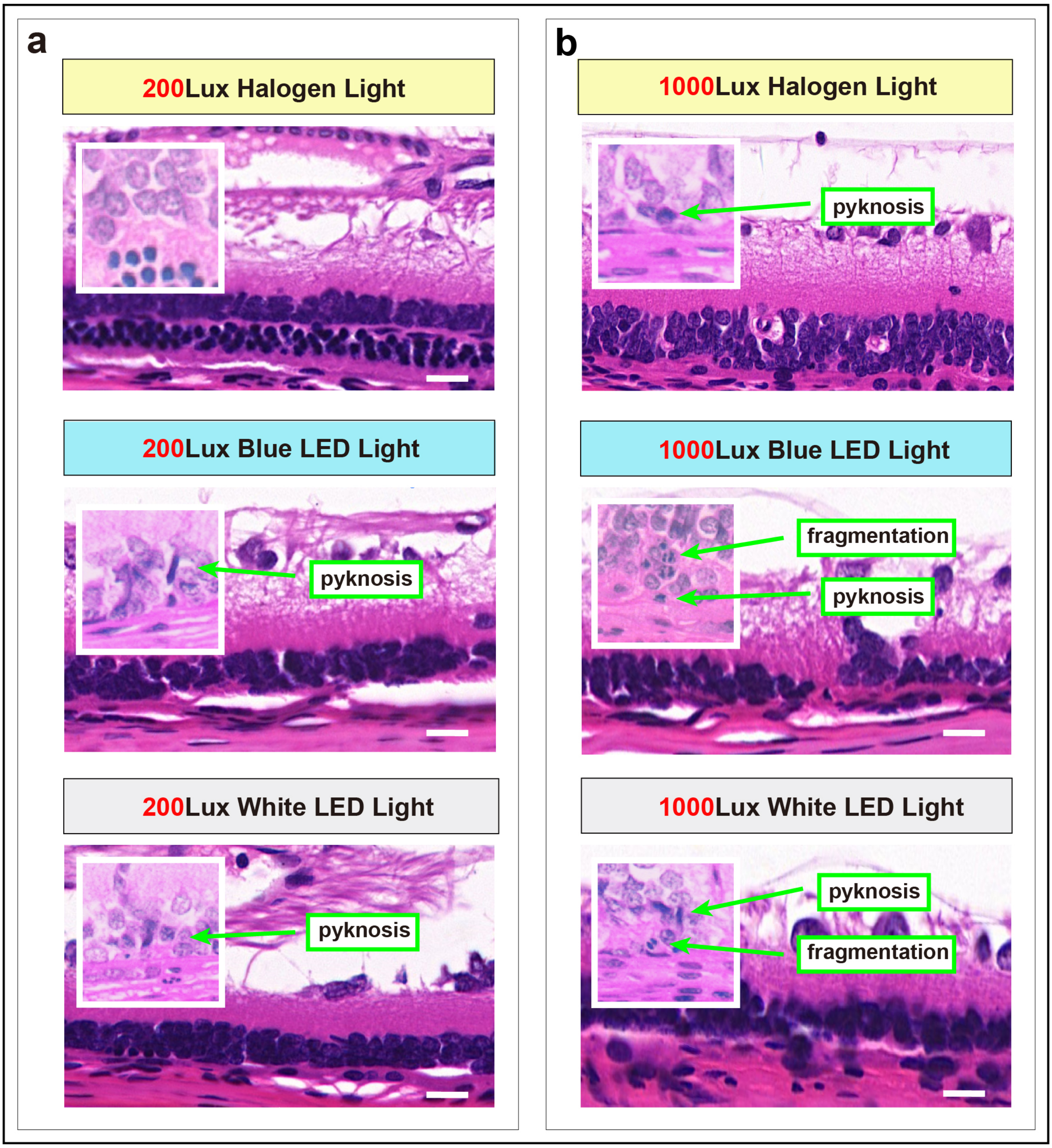

Supplementary Fig. 5 Representative HE images illustrating fragmentation and pyknosis. Scale bars: 50 µm.

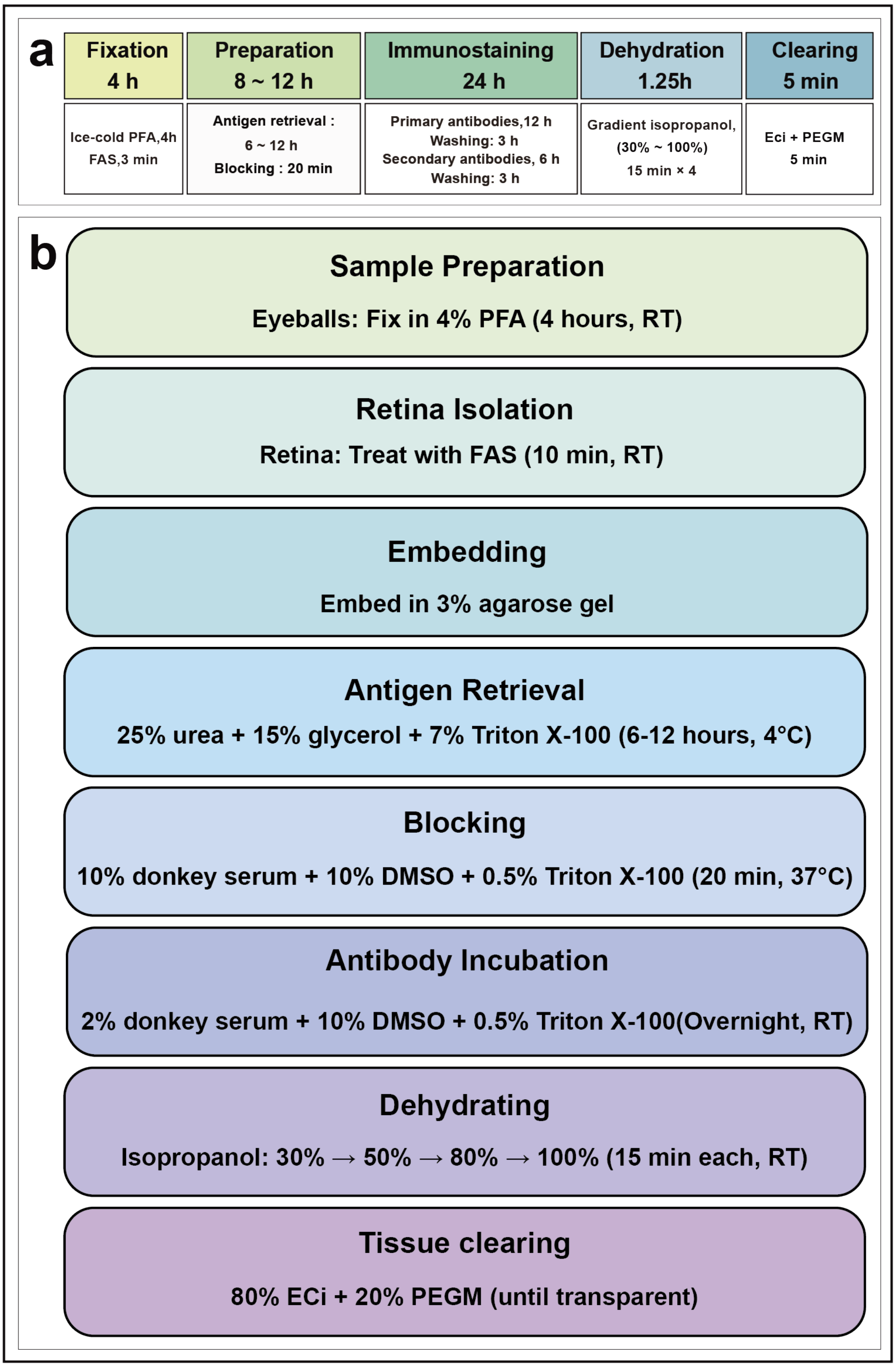

Supplementary Fig. 6 A modified multi-level high-resolution retinal tissue clearing method. a Workflow diagram of a modified multi-level clearing and imaging method on retinal tissue. The figure outlines the time required for each step in each process. **b** A stepwise schematic of modified SUMIC protocol.

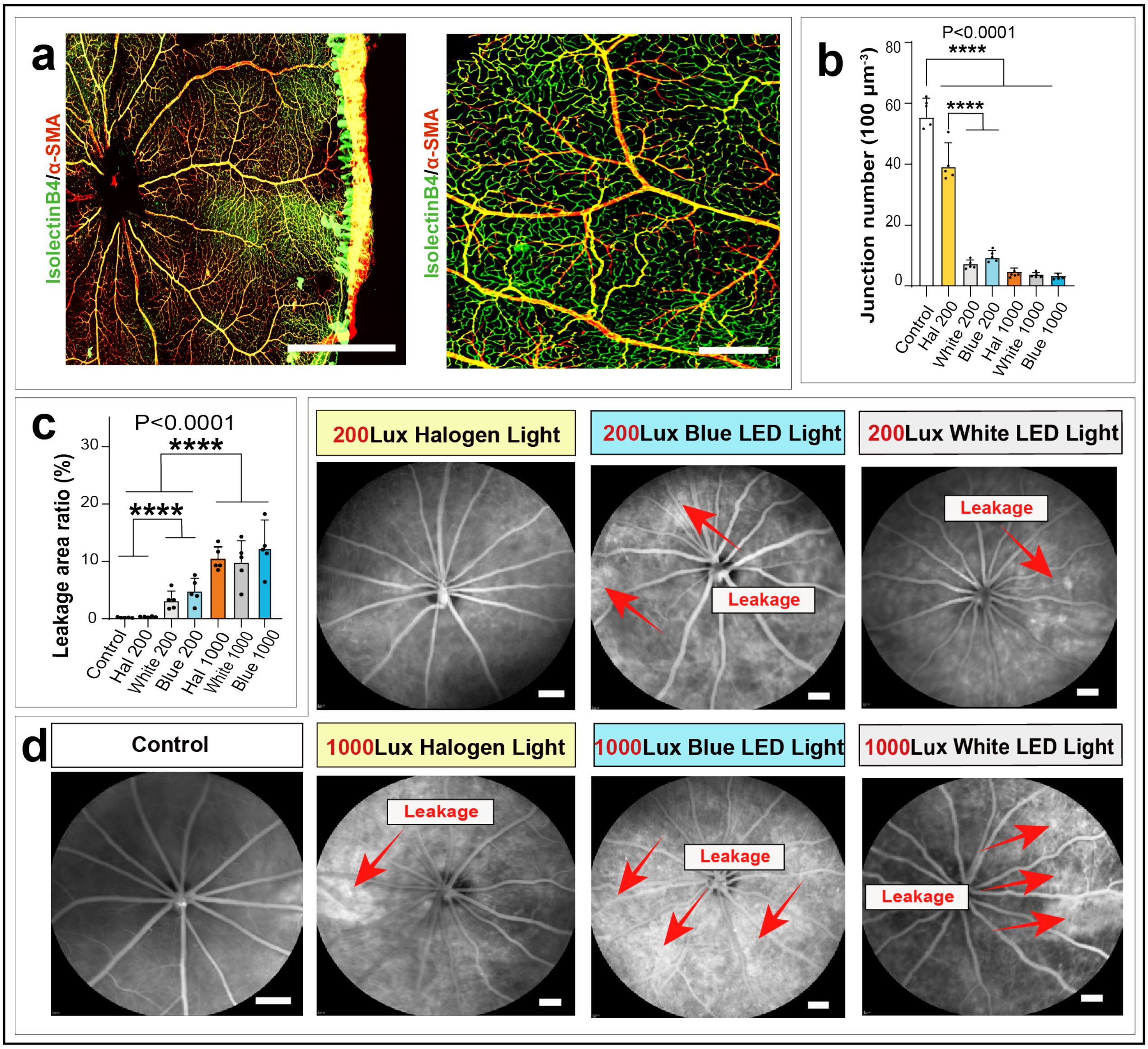

Supplementary Fig. 7 Retinal vessel analysis and perivascular cell changes in response to dental light patterns. a Representative images of retinal vessels in rats stained with α-SMA and Isolectin B4. **b** Quantification of junction number (per 100 μm^3) based on bottom right panels in each group (as detailed in Fig 5a-5d). **c** Quantification of leakage area ratio in different groups. **d** Representative retinal images following injection with sodium fluorescein. Red arrows indicate sodium fluorescein leakage regions. Scale bars: (a) for the left image, 200 μm; for the right image, 50 μm; (d) 1000 μm. Data information: (*n*=5), P value derived from one-way ANOVA test with Student-Newman-Keuls test. ns: not significant; ****P < 0.0001. All data are Mean ± S.D.

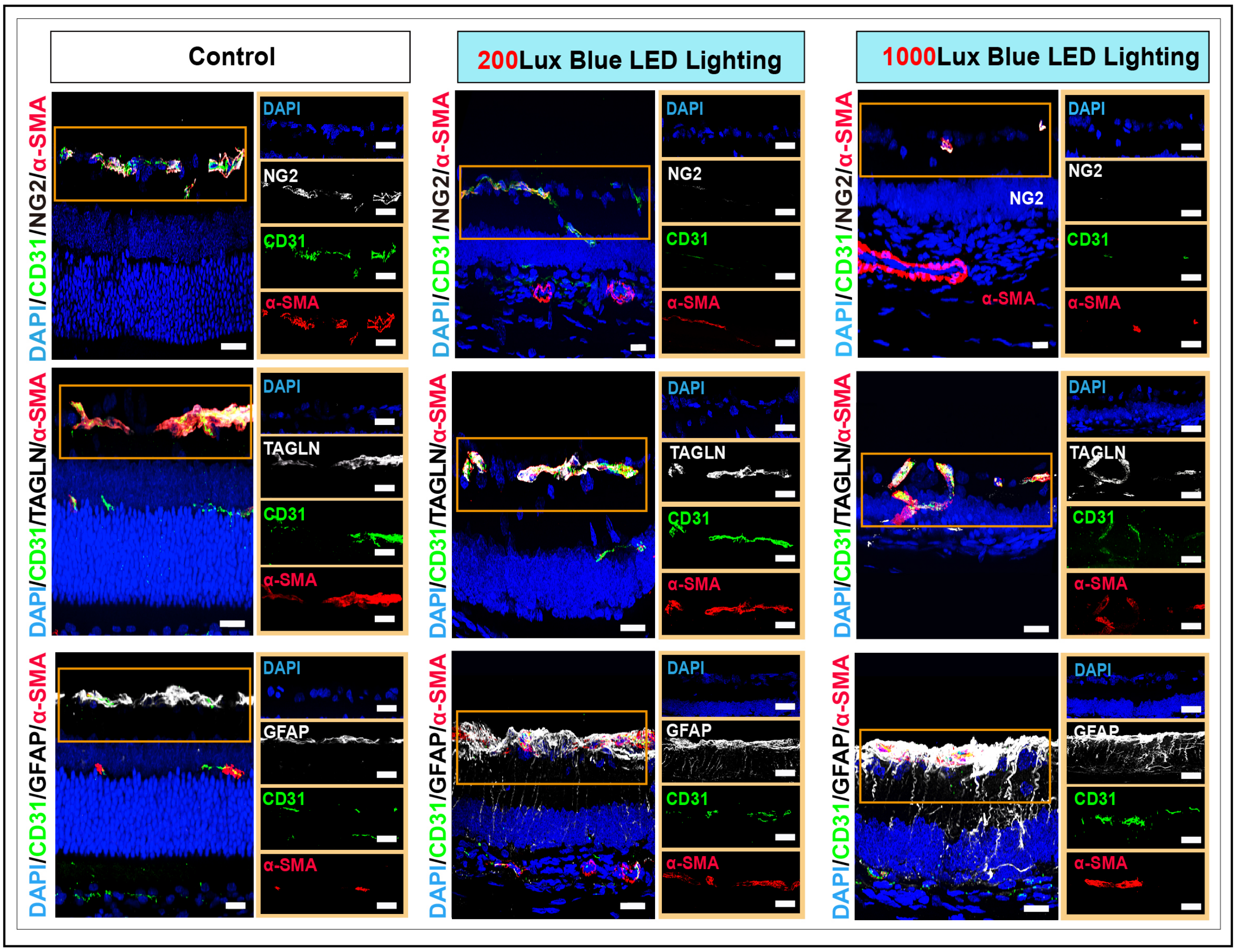

Supplementary Fig. 8 Representative images of the changes in perivascular cells under different light patterns. NG2 to label the pericytes, TAGLN to label the arteries, and GFAP to label the astrocytes. Insets on the right show high magnification of specific areas in the retina. Scale bars: 20 μm.

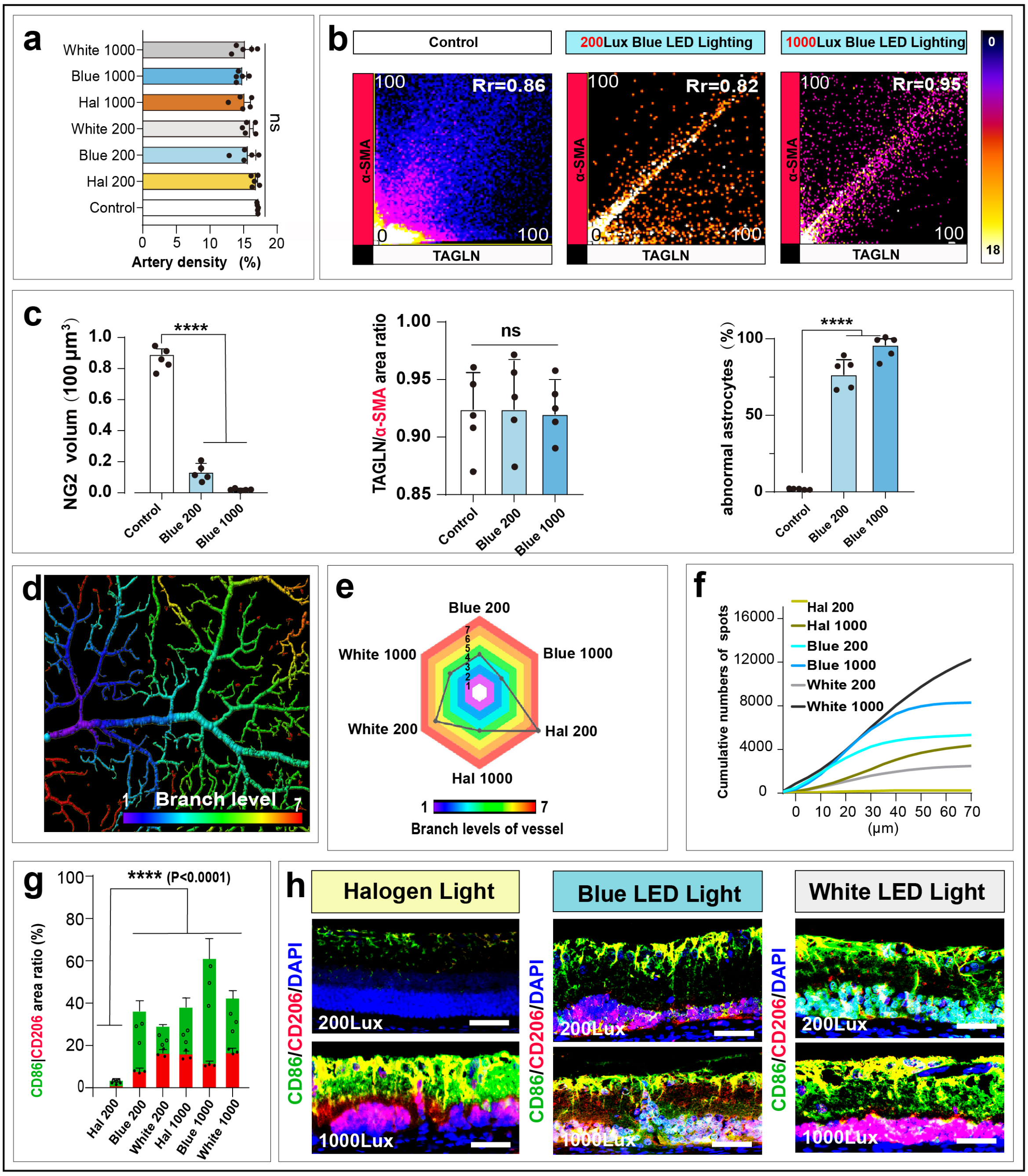

Supplementary Fig. 9 Vessel analysis and Inflammatory response in the retina under the simulated dental lighting patterns. a Quantification of artery density in different groups images (as detailed in Fig. 5). **b** Co-localization statistical graphs of fluorescent signals (TAGLN and α-SMA). **c** Quantifications of the changes in perivascular cells in different groups. **d** Retinal vessels were reconstructed using filament rendering. The color of vessels varies from purple to red according to the calculated branch level. **e** The radar map of the branch level of blood vessels in each group. The color bar varies from purple to red according to the branch level. **f** Quantifications of the cumulative numbers of CD68 at different distance. Each group are displayed as indicated. **g** Quantification of the respective proportions of CD86 and CD206 areas in different groups. **h** Representative images of retinas stained with CD86, CD206 and DAPI. Scale bars: (f) 50 μm.

Data information: (*n*=5), P value derived from one-way ANOVA test with Student-Newman-Keuls test. ns: not significant; ****P < 0.0001. All data are Mean ± S.D. Rr > 0.5 indicates the presence of colocalization.

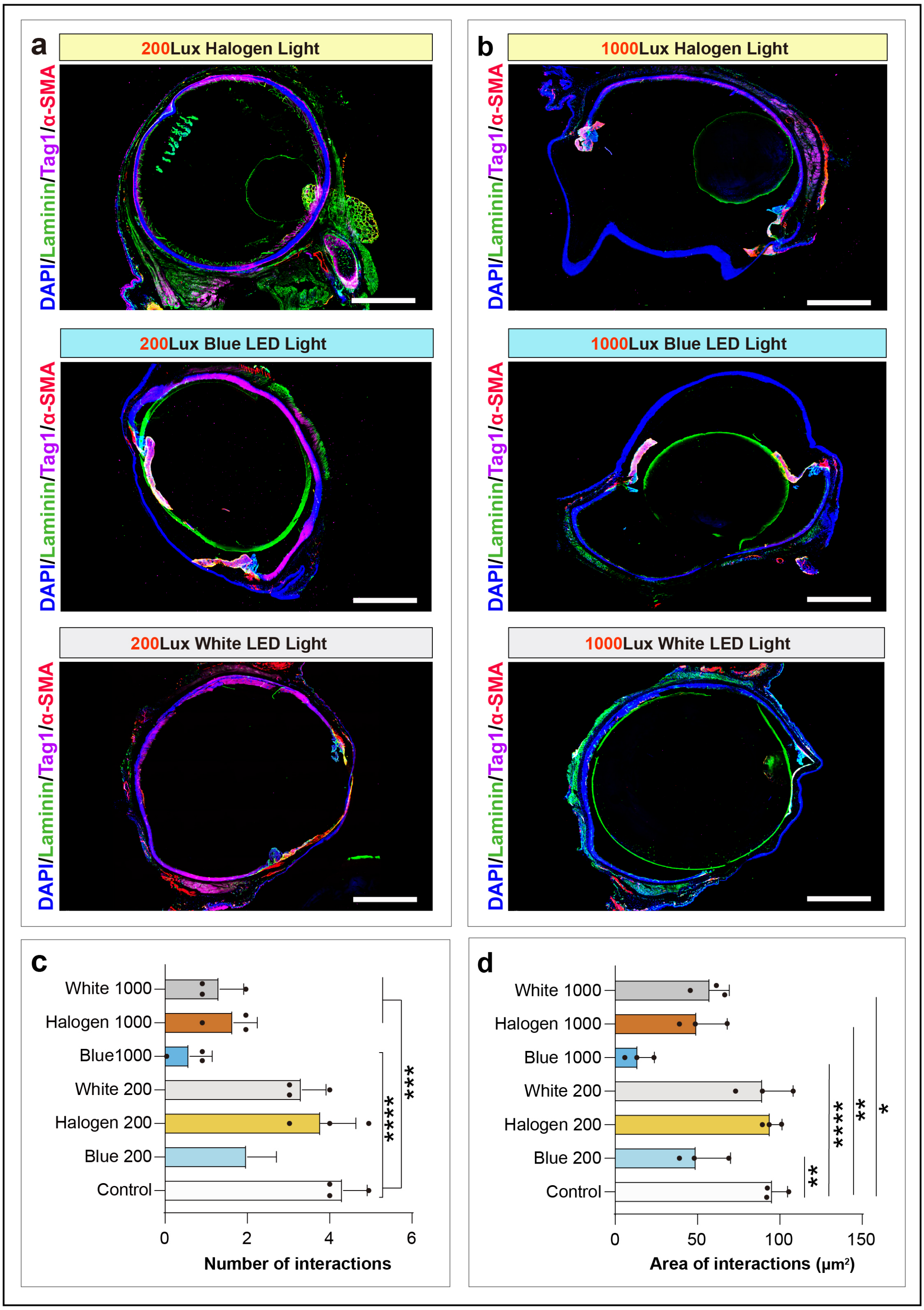

Supplementary Fig. 10 Quantification and visualization of vascular-axon interactions in whole-mount eyeballs. a, b Representative images of whole-mount eyeballs stained with α-SMA, Tag1, Laminin and DAPI. **c** Quantification of numbers of vascular-axon interactions in different groups. **d** Quantification of the area of vascular-axon interactions in different groups. Scale bars: 300 μm. Data information: (*n=3*), P value derived from one-way ANOVA test with Student-Newman-Keuls test. ns: not significant; *P < 0.05, **P < 0.01, ***P < 0.001, ****P < 0.0001. All data are Mean ± S.D.

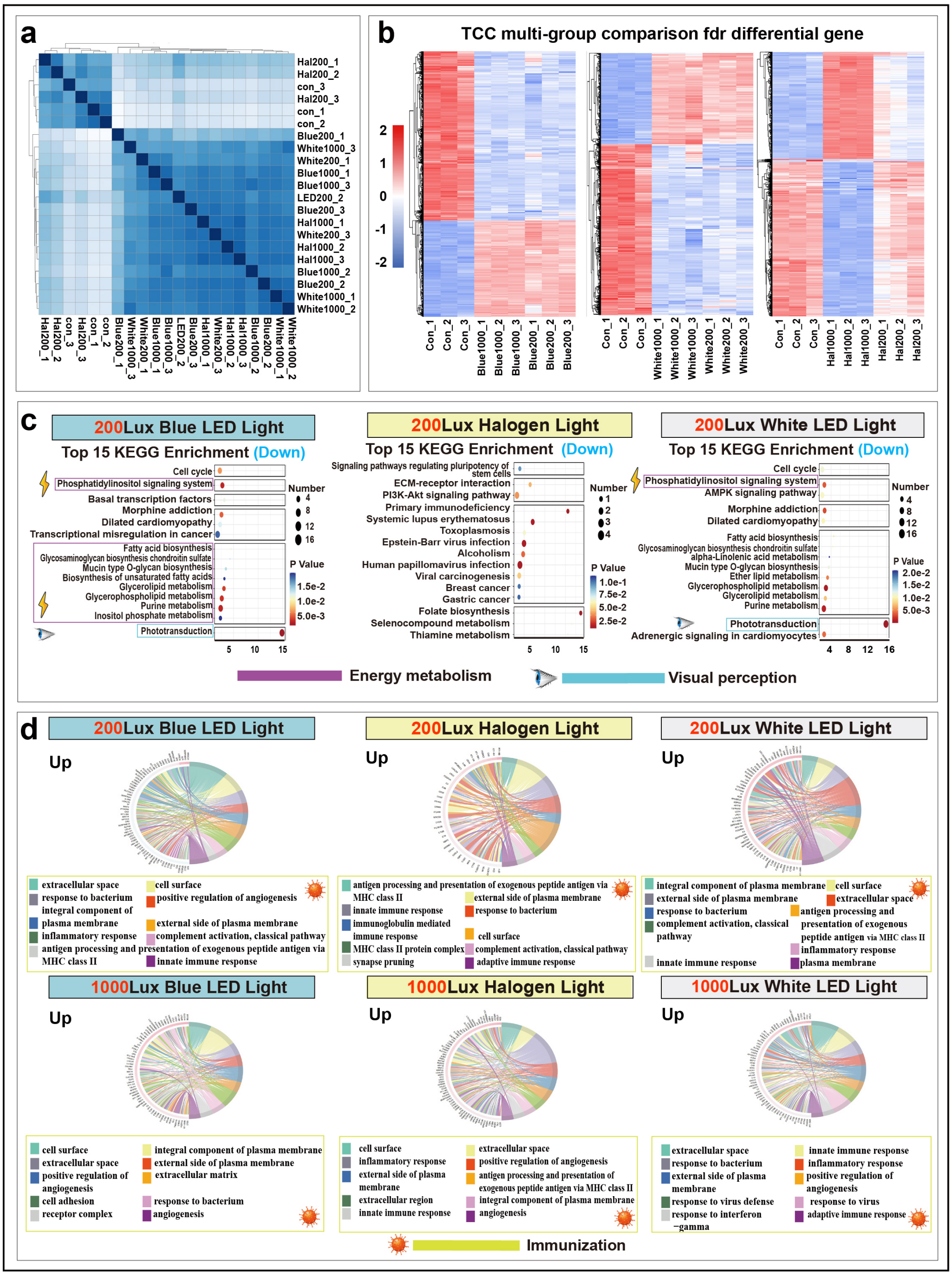

Supplementary Fig. 11 Gene analysis and PCR associated with retinal damage under the simulated dental lighting patterns. a Results of sample-to-sample clustering analysis. The horizontal and vertical axes represent sample names, with colors indicating the magnitude of correlation coefficients. Darker colors correspond to higher correlations between samples. **b** Heatmaps show RNA-seq expression levels of differentially expressed genes between control group and different light sources (FDR-adjusted P value cutoff of <0.01). The color intensity reflects row-scaled normalized log2 (CPM) expression values, with columns corresponding to replicates. Red and blue color intensities denote up-regulated and down-regulated genes, respectively. **c** Top 15 KEGG terms for down-regulated pathways (200 Lux vs. control). Bubble size = gene count; color = P-value (blue to red). **d** Chord diagrams illustrating the top 10 expressed elements within the upregulated entries of Gene Ontology (GO) enrichment analysis for each group. Below, the corresponding entries are displayed.

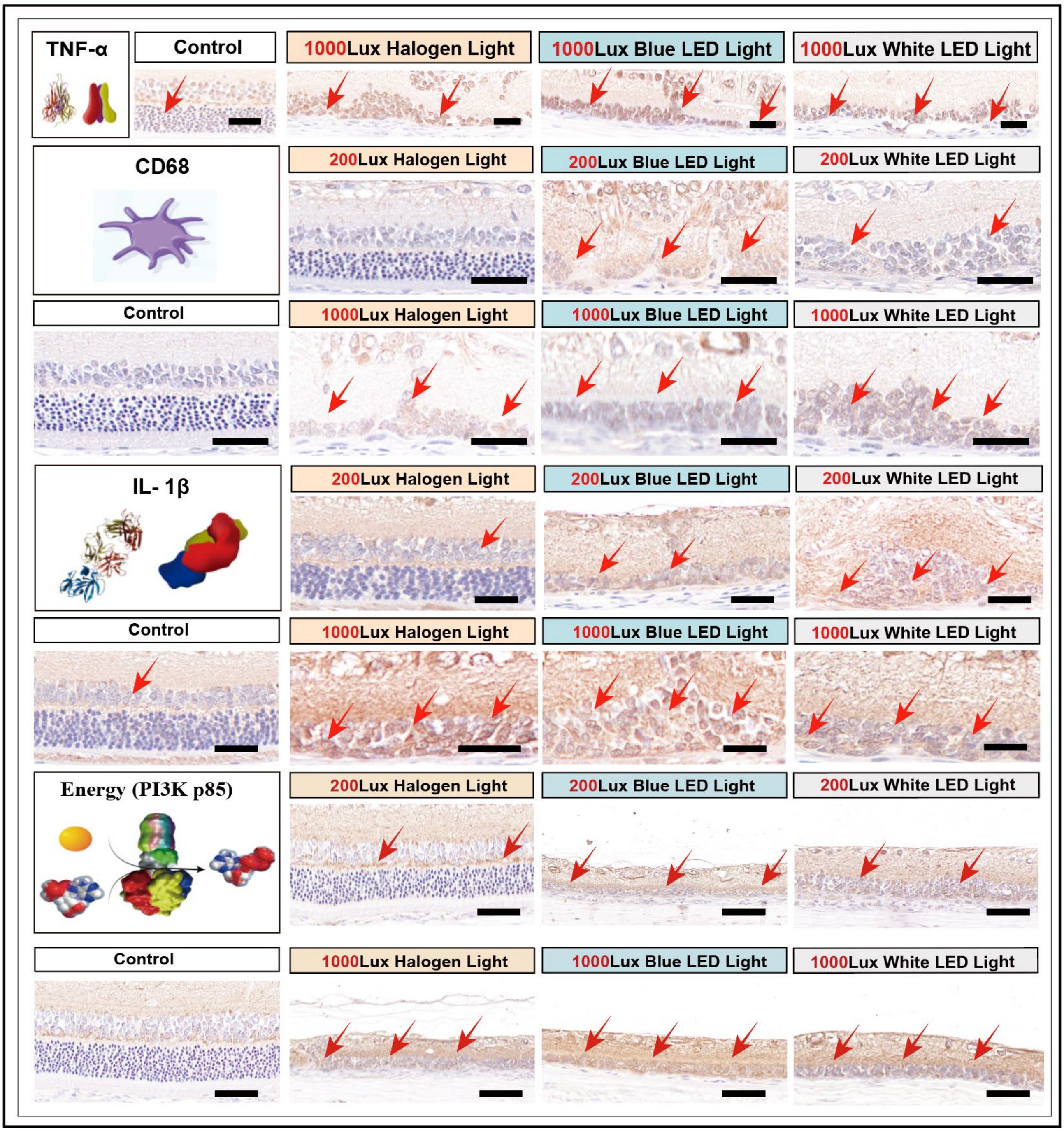

Supplementary Fig. 12 Representative immunohistochemical images showing the stainings on retinal with inflammatory and energy markers. Notable regions of antibody reactivity are delineated by discernible red arrows. Scale bars: 50 μm.

Supplementary Tables

Supplementary Table 1 Characteristics measurement of commonly used dental light sources

Equipment	Light source	Light intensity		Color temperature	Spectral Irradiance	
		indirect	direct		indirect	direct
**Dental micro scope**	Halogen light	212 ± 27 Lux	1152 ± 113 Lux	3229 ± 252 K	2.1 ± 0.4 (mW/m²/nm)	20.3 ± 5.9 (mW/m²/nm)
**Dental curing light**	Blue LED light	241 ± 56 Lux	1552 ± 323 Lux	11085 ± 1620 ~ > 99999 K	16.8 ± 7.3 (mW/m²/nm)	480.3 ± 17.9 (mW/m²/nm)
**Dental chair light**	White LED light	223 ± 38 Lux	1452 ± 313 Lux	5034 ± 381 K	Blue light part: 4.8 ± 0.5 (mW/m²/nm)<br>Yellow light part: 3.8 ± 0.3 (mW/m²/nm)	Blue light part: 24.1 ± 7.0 (mW/m²/nm)<br>Yellow light part: 12.2 ± 4.3 (mW/m²/nm)

Supplementary Table 2 Primary antibodies (dilution ratio, 1:150)

Antibody	Alternative Name	Company	Clone	Cat No.	Host	Reactivity
Actin, α-Smooth Muscle - Cy3™	α-SMA	Sigma-Aldrich	monoclonal	C6198	mouse	human, frog, sheep, chicken, goat, bovine, rat, etc.
CD68	/	Abcam	polyclonal	ab125212	rabbit	mouse, rat
Laminin	/	Abcam	polyclonal	ab11575	rabbit	mouse, human,
Lectin from Bandeiraea simplicifolia	Isolectin B4	Sigma-Aldrich	-	L2895	/	/
RDH5	/	Abcam	polyclonal	ab200197	rabbit	mouse, rat, human
Tag1	CNTN-2	R&D Systems	polyclonal	AF4439	goat	mouse, rat, human,
Ki67	/	Abcam	polyclonal	ab15580	rabbit	mouse, human
CD86	/	Abcam	monoclonal	ab238468	mouse	rat

CD206	/	Invitrogen	polyclonal	PA5-101657	rabbit	mouse, rat, human
IL-1 beta	/	Invitrogen	polyclonal	PA5-46956	goat	rat
TNF alpha	/	Invitrogen	polyclonal	PA1-40281	rabbit	mouse, rat, human
PI 3 Kinase p85	/	Abcam	polyclonal	ab191606	rabbit	mouse, rat, human

Supplementary Table 3 Secondary antibodies (dilution ratio, 1:300)

Antibody	Company	Clone	Cat No.
Donkey anti-Rabbit IgG (H+L) ReadyProbes™ Secondary Antibody, Alexa Fluor 488	Invitrogen	Polyclonal	R37118
Donkey anti-Rabbit IgG (H+L) Highly Cross-Adsorbed Secondary Antibody, Alexa Fluor 647	Invitrogen	Polyclonal	A-31573
Donkey anti-Goat IgG (H+L) Cross-Adsorbed Secondary Antibody, Alexa Fluor 488	Invitrogen	Polyclonal	A-11055
Donkey anti-Goat IgG (H+L) Cross-Adsorbed Secondary Antibody, Alexa Fluor 546	Invitrogen	Polyclonal	A-11056

Supplementary Table 4 Primer Sequences and Base Pairing for PCR Detection

Primer designation	Upstream	Downstream
Il-6	acttccagccagttgccttcttg	tggtctgttgtgggtggtatcctc
Il-1β	agaatcttgaccgtgcgtgcttag	tcatcatcccacgagtcacagagg
Tnf-α	caccacgctcttctgtctactgaac	tgccctcccaccctactttgc
Vcam-1	gtctcatgtcatggcttggtctctg	aagtatgcggcggtgtaattcctg
